# Supplementary material for: Study protocol for evaluation of aid to diagnosis for developmental dysplasia of the hip in general practice: controlled trial randomised by practice
Source: BMJ Open. 2020 Dec 2;10(12):e041837. doi: 10.1136/bmjopen-2020-041837 (PMC7713187; doi:10.1136/bmjopen-2020-041837)
Supplement: Supplementary data [file bmjopen-2020-041837supp001.pdf]

Appendix 1. ROLES AND RESPONSIBILITIES

Funder

The funding body had or has no involvement in study design; collection, management, analysis and interpretation of data; or the decision to submit for publication. The funding body will be informed of any planned publications, and documentation provided.

Sponsor

The sponsor for this trial is Great Ormond Street Hospital for Children. The sponsor is responsible for providing the investigator with the necessary information to conduct the clinical trial, to ensure proper monitoring of the trial and ensuring compliance to ethical bodies and legislation. The sponsor works to the UK Policy Framework for Health and Social Care Research. The sponsor is not involved in aspects of study design, report writing or data analysis. They are the data controller and all data shall return to the sponsor at the end of the trial. A collaboration agreement is in place with all organisations of the co-investigators. Data processing agreements are in place for situations where data will be collected and processed outside of Great Ormond Street Hospital. The sponsor can be contacted by email (research.governance@gosh.nhs.uk) or telephone 0207 905 2249.

Coordinating centre

PRIMENT Clinical Trials Unit is coordinating this trial. A trial management group has been set up within PRIMENT for the monthly monitoring of the trial conduct. It includes the chief investigator, director of the trials unit, programme manager and trial manager. PRIMENT are responsible for overseeing the conduct and progress of the trial.

Steering committee

The steering committee includes an independent chair, two further independent members (one is a biostatistician), sponsor representative, funder representative, chief investigator and two further members of the research team including. The committee will provide overall

supervision of the trial, review analysis plan, recruitment figures and outcome data, and provide recommendations for any appropriate actions for the trial as necessary.

Data monitoring committee

The steering committee will take on the role of the data monitoring committee.

Data Access

All identifiable data will be stored on encrypted servers, UCL Data Safe Haven. Only restricted members of the research team will be able to access this data. De-identified data will be shared with the wider members of the research team.
