# Supplementary material for: Study protocol for evaluation of aid to diagnosis for developmental dysplasia of the hip in general practice: controlled trial randomised by practice
Source: BMJ Open. 2020 Dec 2;10(12):e041837. doi: 10.1136/bmjopen-2020-041837 (PMC7713187; doi:10.1136/bmjopen-2020-041837)
Supplement: Author's manuscript [file bmjopen-2020-041837.draft_revisions.pdf]

BMJ Open

BMJ Open is committed to open peer review. As part of this commitment we make the peer review history of every article we publish publicly available.

When an article is published we post the peer reviewers' comments and the authors' responses online. We also post the versions of the paper that were used during peer review. These are the versions that the peer review comments apply to.

The versions of the paper that follow are the versions that were submitted during the peer review process. They are not the versions of record or the final published versions. They should not be cited or distributed as the published version of this manuscript.

BMJ Open is an open access journal and the full, final, typeset and author-corrected version of record of the manuscript is available on our site with no access controls, subscription charges or pay-per-view fees (http://bmjopen.bmj.com).

If you have any questions on BMJ Open's open peer review process please email info.bmjopen@bmj.com

BMJ Open

Evaluation of aid to diagnosis for developmental dysplasia of the hip in general practice: controlled trial randomised by practice

Journal:	*BMJ Open*
Manuscript ID	bmjopen-2020-041837
Article Type:	Protocol
Date Submitted by the Author:	19-Jun-2020
Complete List of Authors:	Roposch, Andreas; UCL, Institute of Child Health; Great Ormond Street Hospital for Children, Department of Orthopaedic Surgery<br>warsame, kaltuun; University College London Institute of Child Health,<br>Chater, Angel; University of Bedfordshire, Department of Sport Science and Physical<br>Green, Judith; Kings College London<br>Hunter, Rachael; University College London, Research Dept of Primary Care and Population Health<br>Wood, J; University College London, PRIMENT Clinical Trials Unit<br>Freemantle, Nick; University College London, Comprehensive Clinical Trials Unit<br>Nazareth, Irwin; UCL, Primary Care and Population Health
Keywords:	PRIMARY CARE, Paediatric orthopaedics < ORTHOPAEDIC & TRAUMA SURGERY, PAEDIATRICS

SCHOLARONE™
Manuscripts

BMJ

I, the Submitting Author has the right to grant and does grant on behalf of all authors of the Work (as defined in the below author licence), an exclusive licence and/or a non-exclusive licence for contributions from authors who are: i) UK Crown employees; ii) where BMJ has agreed a CC-BY licence shall apply, and/or iii) in accordance with the terms applicable for US Federal Government officers or employees acting as part of their official duties; on a worldwide, perpetual, irrevocable, royalty-free basis to BMJ Publishing Group Ltd ("BMJ") its licensees and where the relevant Journal is co-owned by BMJ to the co-owners of the Journal, to publish the Work in this journal and any other BMJ products and to exploit all rights, as set out in our licence.

The Submitting Author accepts and understands that any supply made under these terms is made by BMJ to the Submitting Author unless you are acting as an employee on behalf of your employer or a postgraduate student of an affiliated institution which is paying any applicable article publishing charge ("APC") for Open Access articles. Where the Submitting Author wishes to make the Work available on an Open Access basis (and intends to pay the relevant APC), the terms of reuse of such Open Access shall be governed by a Creative Commons licence – details of these licences and which Creative Commons licence will apply to this Work are set out in our licence referred to above.

Other than as permitted in any relevant BMJ Author's Self Archiving Policies, I confirm this Work has not been accepted for publication elsewhere, is not being considered for publication elsewhere and does not duplicate material already published. I confirm all authors consent to publication of this Work and authorise the granting of this licence.

For peer review only

Evaluation of aid to diagnosis for developmental dysplasia of the hip in general practice: controlled trial randomised by practice

Andreas Roposch, Kaltuun Warsame, Angel Chater, Judith Green, Rachael Hunter, John Wood, Nicholas Freemantle, Irwin Nazareth

Author Affiliations

Andreas Roposch MD MSc FRCS; Kaltuun Warsame BSc

UCL Great Ormond Street Institute of Child Health

30 Guilford Street, London WC1N 3EH

Angel Chater PhD

Faculty of Education and Sport, University of Bedfordshire

Polhill Avenue, Bedford MK41 9EA

Judith Green PhD

Department of Population Health Sciences, Kings College London

Great Maze Pond, London SE1 1UL

Nicholas Freemantle PhD

Institute of Clinical Trials and Methodology, University College London

90 High Holborn, London WC1V 6LJ

Irwin Nazareth PhD FRCGP; Rachael Hunter MSc; John Wood MSc

Research Department for Primary Care and Population Health, University College London

Rowland Hill Street, London NW3 2PF

Corresponding author: Professor Andreas Roposch, UCL Great Ormond Street Institute of Child Health, 30 Guilford Street, London WC1N 3EH - a.roposch@ucl.ac.uk

Keywords: Developmental dysplasia of the hip, primary care, diagnostic aid

Word count: 3769

For peer review only - http://bmjopen.bmj.com/site/about/guidelines.xhtml

For peer review only

ABSTRACT

Introduction: In the UK, a compulsory '6-week hip check' is performed in primary care for the detection of developmental dysplasia of the hip (DDH). However, missed diagnoses and infants incorrectly labelled with DDH remain a problem, potentially leading to adverse consequences for infants, their families and the NHS. National policy states that infants should be referred to hospital if the 6-week check suggests DDH, though there is no available tool to aid examination or offer guidelines for referral. We developed standardised diagnostic criteria for DDH, based on international Delphi consensus, and a 9-item checklist that has the potential to enable non-experts to diagnose DDH in a manner approaching that of experts.

Methods and Analysis*:* We will conduct a controlled trial randomised by practice that will compare a diagnostic aid against standard care for the hip check. The primary objective is to determine whether an aid to the diagnosis of DDH reduces the number of clinically insignificant referrals from primary care to hospital, and the number of late diagnosed DDH. The trial will include a qualitative process evaluation, an assessment of professional behaviour change and a full health economic evaluation. We will recruit 152 GP practices in England. These will be randomised to conduct the hip checks with use of the study diagnostic aid and/or as per usual practice. The total number of infants seen during a 15-month recruitment period will be 110 per practice. Two years after the 6-week hip check we will measure the number of referred infants that are (1) clinically insignificant for DDH and (2) those that constitute appropriate referrals.

Ethics and Dissemination: This study has approval from the Health Research Authority and the Confidentiality Advisory Group. Results will be published in peer-reviewed academic journals, disseminated to patient organisations and the media.

Registration details: clinicaltrials.gov (NCT04101903; Pre-results).

For peer review only - http://bmjopen.bmj.com/site/about/guidelines.xhtml

ARTICLE SUMMARY

Strengths and limitations

- This is the first trial to evaluate a diagnostic aid for the 6-week check with reference to evaluating both missed and unnecessary referrals to hospital.
- Implementable on existing clinical software used by GPs, the proposed aid will be easy to use.
- A comprehensive process evaluation, using qualitative methods and behaviour change frameworks, will be done alongside to the trial; plus a full health economic evaluation.
- The reliance on existing practice staff to report monthly updates is essential but could pose a risk to timely and complete data collection.
- The collection of identifiable data through site visits across 152 practices will be challenging.

INTRODUCTION

Developmental dysplasia of the hip (DDH) is characterized by varying displacement of the proximal femur from the acetabulum with associated acetabular dysplasia. Dislocation occurs in 1-2/1000 infants per year but milder forms occur in 40-60/1000.[1] Early recognition of disease is associated with better outcomes. It is national policy[2] to examine all infants for the presence of DDH at birth and at age 6-8 weeks in primary care (6-week hip check). If diagnosed within the first 6-8 weeks, splinting of the hips is successful in 85% of cases.[3] Later diagnoses usually will require invasive treatment, with many years of continued monitoring.[4] Late diagnosed DDH is a common cause of medical negligence claims, with increased suffering for affected patients, as well as multi-million costs to the NHS.

For peer review only - http://bmjopen.bmj.com/site/about/guidelines.xhtml

Timely diagnoses of DDH remain a challenge despite the compulsory 6-week check.[5] In one study, the median age at diagnosis was 14 months, with only 40% of infants diagnosed during routine examinations and 60% presenting owing to parental concerns.[6] In another study, 30% of infants were not diagnosed by 12 weeks.[7] Because there is no further compulsory check after that at 6-weeks, it is vital that the 6-week check is effective. Also infants incorrectly identified with "DDH" in primary care and referred to hospital remain a challenge for both families and NHS. This group does not require treatment and reassurance provided in primary care would avoid unnecessary anxiety and inefficient use of hospital resources. Of 1,918 infants referred to hospital from primary care for DDH, only 64 (3%) had DDH[7] but 1,270 (66%) were identified as 'DDH' based on inappropriate criteria,[8] e.g. "crease asymmetry" (n=234) or "click" (n=648). If GPs were able to discriminate better between benign abnormalities requiring reassurance (perhaps with a follow-up in primary care) and findings requiring referrals, outcomes of infants with *and without* DDH would improve.

In prior research we suggested that too many diagnostic criteria[8] and variability in the use of diagnostic criteria among clinicians[9] complicate the task of diagnosing DDH. We developed standardised diagnostic criteria to reduce the variability in assessment and management decisions in infants examined for DDH.[10] The weighted criteria demonstrated validity.[10] In a study of 44 patients referred from GPs to hospital, the weighted criteria demonstrated a positive predictive value of 89% (95% confidence interval 70, 97) and negative predictive value of 76% (95% confidence interval, 50-96).[11] We refined these binary criteria, in form of a checklist with 9 items, for use by GPs during the 6-week hip check and developed a training video, featuring a GP. This checklist and video were refined in a feasibility study which used qualitative methods to explore the acceptability of the format, style and delivery of the intervention (report available on request). The video explains the meaning the diagnostic criteria; it also demonstrates how precisely to elicit the diagnostic criteria. For example, the

For peer review only - http://bmjopen.bmj.com/site/about/guidelines.xhtml

video explains the difference between the Barlow and Ortolani manoeuvres, how to test for a leg length inequality, or how to identify limitations in hip abduction.

Rationale

Current referral patterns suggest there could be considerable health gains from improved diagnostic and referral decisions at the 6-week check.[5] GPs, in a preparatory focus group, identified the need for a 'comprehensive, structured guide' for the 6-week check. Diagnostic aids enable physicians to overcome barriers in diagnostic reasoning[12] by shifting intuitive to analytical aspects of diagnostic reasoning. Decisions made under these circumstances approach normative reasoning and rationality more closely, and are more reliable and safer.[13] Building on our earlier research, we propose to facilitate GP's diagnostic reasoning at the 6-week check by structuring current practice with use of a previously developed diagnostic aid. A diagnostic aid of this kind provides a structured approach to the assessment of infants and offers guidance about referrals.

Objectives

- To determine whether an aid to the diagnosis of DDH reduces the number of clinically insignificant referrals from primary care to hospital, and the number of late diagnosed DDH.
- To determine the cost-effectiveness of this intervention.
- To conduct an integrated qualitative and quantitative process evaluation in order to understand all participants' experience in the trial and with the intervention; study how the intervention is implemented; investigate contextual factors that affect the intervention.

METHODS AND ANALYSIS

The SPIRIT reporting guidelines were used in the preparation of this clinical trial protocol.[14]

Design

For peer review only - http://bmjopen.bmj.com/site/about/guidelines.xhtml

This is a cluster randomised controlled trial of GP practices in England, with randomisation and intervention at practice level but the primary endpoint measured at patient level (Figure 1). An internal pilot will be done in the first 4 months to ascertain accrual rates – we will progress to the main trial if we succeed in recruiting ≥12 practices per month (expected recruitment is ≥21 practices/month for the main trial). Incorporated in the trial are (i) a process evaluation investigating determinants of GPs' referral behaviour and the implementation of the intervention in practice; and (ii) a health economic evaluation in a child's lifetime.

Eligibility and recruitment

GP practices registered in England that carry out 6-week hip checks (at age 42-76 days) and agree (i) to being randomised and (ii) to hospitals releasing data concerning infants they examine in the trial. Ineligible are practices planning to close with 12 months of trial start. We anticipate that ≥110 infants per year will undergo the 6-week hip check in each of the recruited practices. Each practice will recruit infants for a period of 15 months and infants will be followed for 2 years. This trial is planned therefore to run for 44 months across 152 GP practices.

Retention

The proposed trial is not onerous on the practice staff and practices will not be challenged by excessive study-related manoeuvres. Because the primary outcome will be collected using practice and hospital databases only, it is likely that we will attain high levels of follow up. We will send periodic newsletters to GPs and foster regular contacts.

Randomisation

Practices will submit an eligibility form to the clinical trials unit, which will register and randomly allocate (concealed) practices within 48 hours. An independent statistician will coordinate randomisation (allocation ratio 1:1, stratified by practice size).

For peer review only - http://bmjopen.bmj.com/site/about/guidelines.xhtml

Interventions

Control intervention: GPs will assess infant hips following best practice principles; they will be provide a leaflet about the national best practice recommendations.[2]

Experimental intervention: This is a complex intervention comprising a video developed specifically for this trial and a diagnostic aid in form of a 9-item binary checklist. At the time when informed consent is taken, GPs will watch the video. They will then examine all infants in the 6-week check according to best practice principles, but with the addition of the diagnostic aid (implemented electronically to each practice's computer system).

According to national guidelines, the 6-week check shall capture infants at age 6-10 weeks and infants in whom a diagnosis of DDH is considered should see a specialist within 2 weeks.[2] Participating GPs of both intervention arms will be asked to adhere to this policy.

Definitions for the purpose of this trial

'Appropriate referral' denotes a referred hip deemed 'clinically significant', i.e. is treated or monitored (at least one follow-up) by a specialist surgeon. Any ambiguous cases will be reviewed by our Expert Advisory Panel who will assign an ultimate diagnosis in consensus. 'Clinically insignificant' are referred hips resulting in reassurance and discharge from surgeons' clinics (i.e. no treatment or monitoring). 'Late diagnoses' are cases of DDH diagnosed by a specialist surgeon at age 3-24 months.[15;16]

Sample size calculation

We based its calculation on Poisson regression and audit data from the Nottingham area (partially presented in Price et al[7]) and data of 3 years' practice at University College Hospital: a conservative estimate of the number of infants referred as a consequence of the 6-week check within the recruitment period of one year is, on average, 3 per practice. Based on the same data, we estimate that about 1 in 3 (or, on average, 1 per practice) of such referrals is correctly made ('appropriate' referrals, defined above as 'clinically significant', are a

For peer review only

For peer review only - http://bmjopen.bmj.com/site/about/guidelines.xhtml

subset of these ‘correct’ referrals). Thus, on average, we expect 2 ‘incorrect’ referrals per practice. These are the ones we seek to reduce, and the intervention should serve to cut them by 50%. Thus we aim to detect a reduction, on average, from 3 to 2 referrals per practice in the intervention arm. To account for correlation within practices, which can also be thought of as overdispersion relative to the underlying Poisson variation, we assumed a between-practice component of variance of 20% of the average Poisson ‘counting’ variance per practice. For 90% statistical power and testing 2-sided at p=0.05, we need 76 practices per group (15% of this total comprises a safety margin to allow for potential challenges). Average referral rates from the Nottingham (2.8%) and University College Hospital data (2.7%) are very consistent, and suggest a target of about 110 children per practice per year to undergo the 6-week check.

Primary trial endpoint

Number of referred infants that are considered 'clinically insignificant' – this is a measure of the clinical importance of referrals in the 2-week hip pathway. We chose this outcome because it is extremely relevant clinically and one for which it is possible to power the trial.[2] An intervention could be successful at achieving this outcome whilst missing infants who do have DDH; we thus specified a *principal secondary endpoint*. False negatives are also important but, because of their rarity, would not be an efficient endpoint on which to power the trial (and would thus be outside ICH E9,[17] which recommends that the primary outcome should be both clinically and statistically convincing). A research assistant will retrieve these data from respective hospital electronic systems by conducting site visits to all secondary care facilities to which GPs refer infants, starting 6 weeks after the last patient enters the trial.

Secondary trial endpoints (Table 1)

Infant level

For peer review only - http://bmjopen.bmj.com/site/about/guidelines.xhtml

• Number of appropriate referrals per practice (principal secondary endpoint): for all infants referred as a consequence of the 6-week check, a researcher will collect, using unique NHS patient numbers, and categorise the appropriateness of referrals by a practice with respective hospital databases using a standardised taxonomy, blind to practice random allocation. S/he will conduct site visits to all such hospitals starting 6 weeks after the last patient entered the trial.

• Number of late diagnoses: We will employ deterministic methods of data linkage. Using unique NHS numbers of any recruited infant, we will obtain from Health & Social Care Information Centre the corresponding Health Episodes Statistic (HES) identifiers. HES is a data warehouse that includes all hospital admissions and outpatients' visits occurring in all hospitals in England. In HES we will establish whether any infant in the trial was admitted to a UK hospital as a result of DDH using relevant ICD-10 codes and OPCS-4 codes.[18] We will extract, for the whole trial period, the full hospital history for all infants in the trial (in and out patient episodes), collate all episodes into a combined exploratory analysis view, collate interactions, and allocate outcomes to practice groups to facilitate outcome analyses.[19] These data will be compared with the data collected by the researcher (see above); the use of these 2 strategies will enhance the robustness of data.

• Consequences of late diagnoses: Using data collected above we will record nature and frequency of such consequences (nature of treatment, length of hospital stay, frequency of secondary care contacts).

• Health-related quality of life (parental proxy report): Child-Health-Utility-9D.[20]

Process level

• Volume of referrals: Total number of patients referred to secondary care during the trial period. This variable will be collected prospectively at practice level by the participating GP and forwarded monthly to the clinical trial unit.

For peer review only - http://bmjopen.bmj.com/site/about/guidelines.xhtml

• Timeliness of referrals: In infants referred to secondary care, we will measure the days from referral issued to hospital appointment (collected in the same way as primary endpoint). This process measure will inform about target wait times[2] achieved.

Clinician level

• Confidence and attitudes of GP and secondary care clinicians towards the diagnostic aid: will be assessed 12 weeks after trial set-up and at trial completion using a modified measure based on the Theory of Planned Behaviour.[21]

• Implementation issues and acceptability of diagnostic aid among GPs and secondary care providers: we will conduct qualitative research at the trial end to elicit this information.

• Use of diagnostic aid and acceptability of intervention: a self-administered questionnaire to evaluate the use of the aid will be posted to all the GPs in the intervention group; also direct observations and interviews will ascertain this outcome.

Parent/carer level (collected from one in ten parents/carers)

• General worry: State-Trait Anxiety Inventory 6-items short form.[22]

• DDH-related worry: Infant Hip Worries Inventory.[23]

• Satisfaction with trial: dimensions of care items from EUROPEP.[24]

For peer review only

Table 1. Assessments at different time points

	Baseline	At 6-week hip check	Within 2 weeks of check	3-month follow-up	15-month follow-up	2-year follow-up
Clinician-level						
Theoretical Domains Framework questionnaire	X		X	X	X	X
GP characteristics questionnaire	X					
Fidelity questionnaire			X	X	X	X
Non-participant observations		X				
Semi-structured interviews			X		X	
Parent/carer-level						
State Trait Anxiety Inventory 6 item short form			X			
Infant Hip Worries Inventory			X			
EUROPEP			X			
Non-participant observations		X				
Semi-structured interviews			X			
Out-of-pocket-costs questionnaire			X			
EuroQol 5 Dimension Scale (EQ-5D)			X			X
Child Health Utility 9D (CHU 9D)						X
Willingness-to-pay questionnaire					X	

For peer review only

Statistical analysis

Primary endpoint: We will compare the randomised practices using Poisson mixed models, accounting for extra *Poissonian* variability by including random intercept terms for practices. The response variable will be the number of clinically insignificant referrals from each practice with an offset in the linear model of the log(e) total number of children checked in each practice, to account for differences in practice size and constitution. The random effect at the practice level will account for overdispersion.

Principal secondary endpoint: This will be analysed as above; however, counts here are expected to be lower. Should they be insufficient to support the full model, the model will be simplified by (e.g. use of additive variance component term rather than a generalised random intercept term to address overdispersion). We will describe the planned statistical analysis and its adaptation where there are difficulties in achieving convergence in a pre-specified statistical analysis plan.

Late diagnoses: As we anticipate small numbers of events in each randomised comparison we may not be in a position to account for practices using random intercept terms. Where this is the case we will report the total numbers over a minimum 2-year observation and compare the overall group scores using Fishers exact test.

Quantitative outcome measures: We will summarize scores by instrument, accounting for practices and report difference in group means for each treatment arm, using appropriate transformation where necessary. We expect mean scores to be lower in the intervention arm for State-Trait Anxiety Inventory and Infant Hip Worries Inventory, but higher or equal for EUROPEP.

Missing data: For the primary endpoint and principal secondary endpoint the data collection methods should identify qualifying episodes. Because of the nature of these data the conventional concept of missingness does not directly apply (eg we will not have individually randomised subjects who cannot be followed up). However if a practice withdraws from the trial we will explore the consequences of this action by assuming a poor outcome among that practice if in the intervention

For peer review only

group and a good outcome if in the control condition, to identify the potential consequences of their withdrawal. Complete case analyses will be conducted for secondary outcomes. If there is a mismatch between practices in the two treatment conditions we will consider undertaking joint models examining simultaneously the binomial of missingness and the outcome measure of interest.

Health economics

Cost analysis

We will analyse the cost associated with the intervention compared with usual practice for the entire trial period, and examine costs from the perspective of the NHS and of families. The cost components included in main analysis are: cost of 6-week in both intervention arms; any subsequent referrals, diagnostic tests and treatment. We will collect costs about GP time, which we will multiply by unit costs from routine sources.[25]

Within-trial cost-effectiveness analysis

With the costs described above we will produce a dataset of patient-level within-trial costs and outcomes. We will calculate the incremental cost per clinically insignificant referral avoided and the incremental cost per late diagnosis avoided. Using bootstrapping of the mean cost and outcomes differences, we will estimate confidence intervals around the incremental cost-effectiveness ratios.[26] With the bootstrap replications we will construct a cost-effectiveness acceptability curve to show the probability that the aid is cost-effective for different values of NHS willingness to pay for outcomes. We will perform deterministic sensitivity analyses.

Long-run cost utility analysis

We will use several measures to evaluate the lifetime cost-effectiveness of the intervention. We will ask 20 carers of infants aged 2-4 years to complete (1) on behalf of their children the Child-Health-utility-9D[20] and (2) for themselves the EQ-5D-5L,[27] both measure health-related quality of life.

Cost-benefit analysis

For peer review only - http://bmjopen.bmj.com/site/about/guidelines.xhtml

With data from the trial about the impact of the intervention on appropriate referrals, we will calculate the monetary value that parents place on the intervention using willingness-to-pay methodology.[28] This will provide an estimate of the monetary value of the additional benefits (positive/negative) of the intervention. We will calculate the net benefit of the intervention by subtracting the incremental cost of the aid, as calculated above from the trial data, from the monetary value of the additional benefit. Following recruitment of the last infant in the trial, we will recruit 200 carers of infants undergoing the 6-week check from trial-participating practices. These will be 100 carers whose infants will be referred to secondary care as a consequence of the 6-week check and 100 who will not. They will complete a self-report questionnaire that utilised several techniques[28] to elicit willingness-to-pay values. We will calculate willingness-to-pay values for the whole sample and test for variations based on socio-demographic groups and referral to secondary care.

Integrated qualitative and quantitative process evaluation

This workstream will explore the implementation, adherence to protocol, receipt and setting of the intervention. We will examine the views of all groups of participants on the intervention; study how the intervention is implemented; investigate contextual factors that affect the intervention; and study how effects vary in subgroups of GPs. These data will help in understanding how, for whom and why the trial had effects and the extent to which outcomes result from issues of trial fidelity and implementation. We will collect process data from all 152 sites including clinician and carer outcomes. We will conduct alongside the trial non-participant observation and semi-structured interviews. We will include a purposive sample of 10 practices for the qualitative study, interviewing 4-5 participants in each (e.g. GP, carers, hospital consultant), resulting in 40-50 interviews. This sample will include a small number of practices in the control arm (for comparative purposes), and a range of intervention practices to include different locations, practice sizes and types. We will analyse process data before outcome data to avoid bias in interpretation.[29] Interviews will be audio-

For peer review only - http://bmjopen.bmj.com/site/about/guidelines.xhtml

recorded and transcribed, data from observations will be recorded contemporaneously using a template. Data from process outcomes, interviews (transcripts) and observations will be analysed from the perspective of both behaviour change theory[30] and normalization process theory.[31]

Strategies to mitigate potential bias

Since this effectiveness trial will test whether the intervention can work under usual circumstances, we will rely on paediatric orthopaedic surgeons in determining the ultimate diagnosis of DDH. Variations in the surgeons' diagnostic accuracy are inevitable hence the need for a randomised study. We will perform analyses by surgeon (or hospital) to quantify this variation. Blinding of GPs, practice staff, carers is impossible; however, most such outcomes will be assessed with validated questionnaires. Primary and principal secondary endpoints will be collected by an independent researcher blinded to treatment allocation. In case an infant is referred to hip ultrasound without an orthopaedic consultation, a trial-appointed advisory panel shall review the scan blinded and according to standard methods[32] to avoid reporting bias. There is a risk for verification bias – while our trial includes a 2-year follow up to capture late presenting DDH, we cannot rule out that some infants with DDH will remain undiagnosed within this period, thus underestimating the number of late diagnosed DDH. However, the 2-year mark has previously been found to be a robust outcome.[33]

Patient and public involvement

We developed this protocol with carers of children with DDH and the founding director of 'Steps', a charity supporting patients with lower limb disorders. We discussed the need for the trial and trial procedures and conduct with staff members of GP practices. Our established patient and public involvement group has reviewed and commented on this protocol and will periodically review, support and advise on the conduct of the trial.

Trial and data management (Appendix 1)

The trial will be run through PRIMENT Clinical Trials Unit and conducted in accordance with established quality management systems and standardised operating procedures. All data will be

For peer review only - http://bmjopen.bmj.com/site/about/guidelines.xhtml

handled in accordance with the UK Data Protection Act 2018. We will use double data entry at all states. All analyses will be conducted blinded to allocation groups.

Ethics and dissemination

Protocol version 2.0 (12/11/2019) received approval from the Leicester Central Research Ethics Committee (19/EM/0317) and Health Research Authority Confidentiality Advisory Group (19/CAG/0198). It is registered at clinicaltrials.gov (NCT04101903). The results will be published in peer-reviewed academic journals, disseminated to patient organisations and the media.

Discussion

This randomised clinical trial is part of a programme of research to improve the diagnosis of DDH: consensus-based diagnostic criteria were established in prior research, tailored for use in primary care in form of a 'checklist', supplemented by a video specifically designed to explain to GPs how to use the tool at bedside. There has only been one randomised clinical trial on the topic of DDH in the UK[33] but it explored the use of ultrasound screening – our trial focusses on the compulsory clinical assessment conducted by GPs. Because the intervention tested in this trial is based on consensus of clinical experts, there is a risk that the opinions of experts change as clinical knowledge evolves. However, the criteria of the diagnostic aid have been in use for decades and will likely not lose relevance in the foreseeable future. The collection of outcome data from various hospitals connected to GP practices will be challenging; use of national health services databases should mitigate this challenge. This trial has the potential to improve the compulsory 6-week hip check with use of a relatively simple intervention. It will also provide an understanding of the cost effectiveness of the intervention in a whole lifetime horizon of a 6-week old. If successful, the intervention can be rolled out to clinical services relatively easily and at low costs.

Author contributions

For peer review only - http://bmjopen.bmj.com/site/about/guidelines.xhtml

AR and KW wrote the first draft of the paper. All authors conceived the study and revised the manuscript for relevant scientific content. NF, JW specifically revised the statistical content. All authors approved the final version the manuscript.

Funding statement

This paper presents independent research funded by the National Institute for Health Research (NIHR) under its Programme Grant for Applied Research funding stream (RP-PG-0616-20006). The views expressed are those of the authors and not necessarily those of the NIHR or the Department of Health and Social Care.

Competing interest statement

None

References

(1) Bialik V, Bialik GM, Blazer S, Sujov P, Wiener F, Berant M. Developmental dysplasia of the hip: a new approach to incidence. *Pediatrics* 1999; 103(1):93-99.

(2) Public Health England. Newborn and infant physical examination (NIPE) sceening handbook. www.gov.uk/government/publications/newborn-and-infant-physical-examination-programme-handbook/newborn-and-infant-physical-examination-screening-programme-handbook . 27-8-2019.

(3) Clarke NM, Reading IC, Corbin C, Taylor CC, Bochmann T. Twenty years experience of selective secondary ultrasound screening for congenital dislocation of the hip. *Arch Dis Child* 2012; 97(5):423-429.

(4) NJR Editorial Board. 10th Annual report: national joint registry for England, Wales and Northern Ireland. 2013.

(5) Davies R, Talbot C, Paton R. Evaluation of primary care 6- to 8-week hip check for diagnosis of developmental dysplasia of the hip: a 15-year observational cohort study. *Br J Gen Pract* 2020; 70(693):e230-e235.

(6) Sanghrajka AP, Murnaghan CF, Shekkeris A, Eastwood DM. Open reduction for developmental dysplasia of the hip: failures of screening or failures of treatment? *Ann R Coll Surg Engl* 2013; 95(2):113-117.

For peer review only - http://bmjopen.bmj.com/site/about/guidelines.xhtml

(7) Price KR, Dove R, Hunter JB. Current screening recommendations for developmental dysplasia of the hip may lead to an increase in open reduction. *Bone Joint J* 2013; 95-B(6):846-850.

(8) Roposch A, Liu LQ, Hefti F, Clarke NM, Wedge JH. Standardized diagnostic criteria for developmental dysplasia of the hip in early infancy. *Clin Orthop Relat Res* 2011; 469(12):3451-3461.

(9) Roposch A, Liu LQ, Protopapa E. Variations in the use of diagnostic criteria for developmental dysplasia of the hip. *Clin Orthop Relat Res* 2013; 471(6):1946-1954.

(10) Roposch A, Protopapa E, Cortina-Borja M. Weighted diagnostic criteria for developmental dysplasia of the hip. *J Pediatrics* 2014; 165:1236-1240.

(11) Roposch A, Protopapa E. Testing newly developed standardized diagnostic criteria for DDH in consecutive patients referred to a paediatric orthopaedic unit. *Pediatric Orthopedic Society of North America* 2013.

(12) El-Kareh R, Hasan O, Schiff GD. Use of health information technology to reduce diagnostic errors. *BMJ Qual Saf* 2013; 22 Suppl 2:ii40-ii51.

(13) Croskerry P. Clinical cognition and diagnostic error: applications of a dual process model of reasoning. *Adv Health Sci Educ Theory Pract* 2009; 14 Suppl 1:27-35.

(14) Chan AW, Tetzlaff JM, Altman DG, Laupacis A, Gotzsche PC, Krleza-Jeric K et al. SPIRIT 2013 statement: defining standard protocol items for clinical trials. *Ann Intern Med* 2013; 158(3):200-207.

(15) Woolacott NF, Puhan MA, Steurer J, Kleijnen J. Ultrasonography in screening for developmental dysplasia of the hip in newborns: systematic review. *BMJ* 2005; 330(7505):1413.

(16) Clinical practice guideline: early detection of developmental dysplasia of the hip. Committee on Quality Improvement, Subcommittee on Developmental Dysplasia of the Hip. American Academy of Pediatrics. *Pediatrics* 2000; 105(4 Pt 1):896-905.

(17) ICH Steering Committee. ICH harmonised tripartite guideline: Statistical principles for clinical trials. https://database.ich.org/sites/default/files/E9_Guideline . 5-2-1998.

(18) NHS Digital. National clinical coding standards OPCS-2 (2018). Norwich, England: TSO Shop; 2018.

(19) MacArthur C, Jolly K, Ingram L, Freemantle N, Dennis CL, Hamburger R et al. Antenatal peer support workers and initiation of breast feeding: cluster randomised controlled trial. *BMJ* 2009; 338:b131.

(20) Stevens K. Assessing the performance of a new generic measure of health-related quality of life for children and refining it for use in health state valuation. *Appl Health Econ Health Policy* 2011; 9(3):157-169.

For peer review only - http://bmjopen.bmj.com/site/about/guidelines.xhtml

(21) Powell AA, Bloomfield HE, Burgess DJ, Wilt TJ, Partin MR. A conceptual framework for understanding and reducing overuse by primary care providers. *Med Care Res Rev* 2013; 70(5):451-472.

(22) Marteau TM, Bekker H. The development of a six-item short-form of the state scale of the Spielberger State-Trait Anxiety Inventory (STAI). *Br J Clin Psychol* 1992; 31 (Pt 3):301-306.

(23) Gardner F, Dezateux C, Elbourne D, Gray A, King A, Quinn A et al. The hip trial: psychosocial consequences for mothers of using ultrasound to manage infants with developmental hip dysplasia. *Arch Dis Child Fetal Neonatal Ed* 2005; 90(1):F17-F24.

(24) Wensing M. EUROPEP 2006: Revised EUROPEP instrument and user manual. www.equip.ch/files/25/europep_2006rapport.pdf 6-6-2014.

(25) Department of Health. Department of Health. National Schedule of Reference Costs - Year 2011-12 - NHS Trusts and NHS Foundation Trusts: NHS own costs. 2015.

(26) Briggs AH, Wonderling DE, Mooney CZ. Pulling cost-effectiveness analysis up by its bootstraps: a non-parametric approach to confidence interval estimation. *Health Econ* 1997; 6(4):327-340.

(27) Herdman M, Gudex C, Lloyd A, Janssen M, Kind P, Parkin D et al. Development and preliminary testing of the new five-level version of EQ-5D (EQ-5D-5L). *Qual Life Res* 2011; 20(10):1727-1736.

(28) McIntosh E. Using discrete choice experiments within a cost-benefit analysis framework: some considerations. *Pharmacoeconomics* 2006; 24(9):855-868.

(29) Oakley A, Strange V, Bonell C, Allen E, Stephenson J. Process evaluation in randomised controlled trials of complex interventions. *BMJ* 2006; 332(7538):413-416.

(30) Michie S, Richardson M, Johnston M, Abraham C, Francis J, Hardeman W et al. The behavior change technique taxonomy (v1) of 93 hierarchically clustered techniques: building an international consensus for the reporting of behavior change interventions. *Ann Behav Med* 2013; 46(1):81-95.

(31) Murray E, Treweek S, Pope C, MacFarlane A, Ballini L, Dowrick C et al. Normalisation process theory: a framework for developing, evaluating and implementing complex interventions. *BMC Med* 2010; 8:63.

(32) Roposch A, Moreau NM, Uleryk E, Doria AS. Developmental dysplasia of the hip: quality of reporting of diagnostic accuracy for US. *Radiology* 2006; 241(3):854-860.

(33) Elbourne D, Dezateux C, Arthur R, Clarke NMP, Gray A, King A et al. Ultrasonography in the diagnosis and management of developmental hip dysplasia (UK Hip Trial): clinical and economic results of a multicentre randomised controlled trial. *Lancet* 2002; 360(9350):2009-2017.

For peer review only - http://bmjopen.bmj.com/site/about/guidelines.xhtml

Figure legends

Figure 1. Flowchart demonstrating pathways and principal endpoints and their collection.

For peer review only

For peer review only - http://bmjopen.bmj.com/site/about/guidelines.xhtml

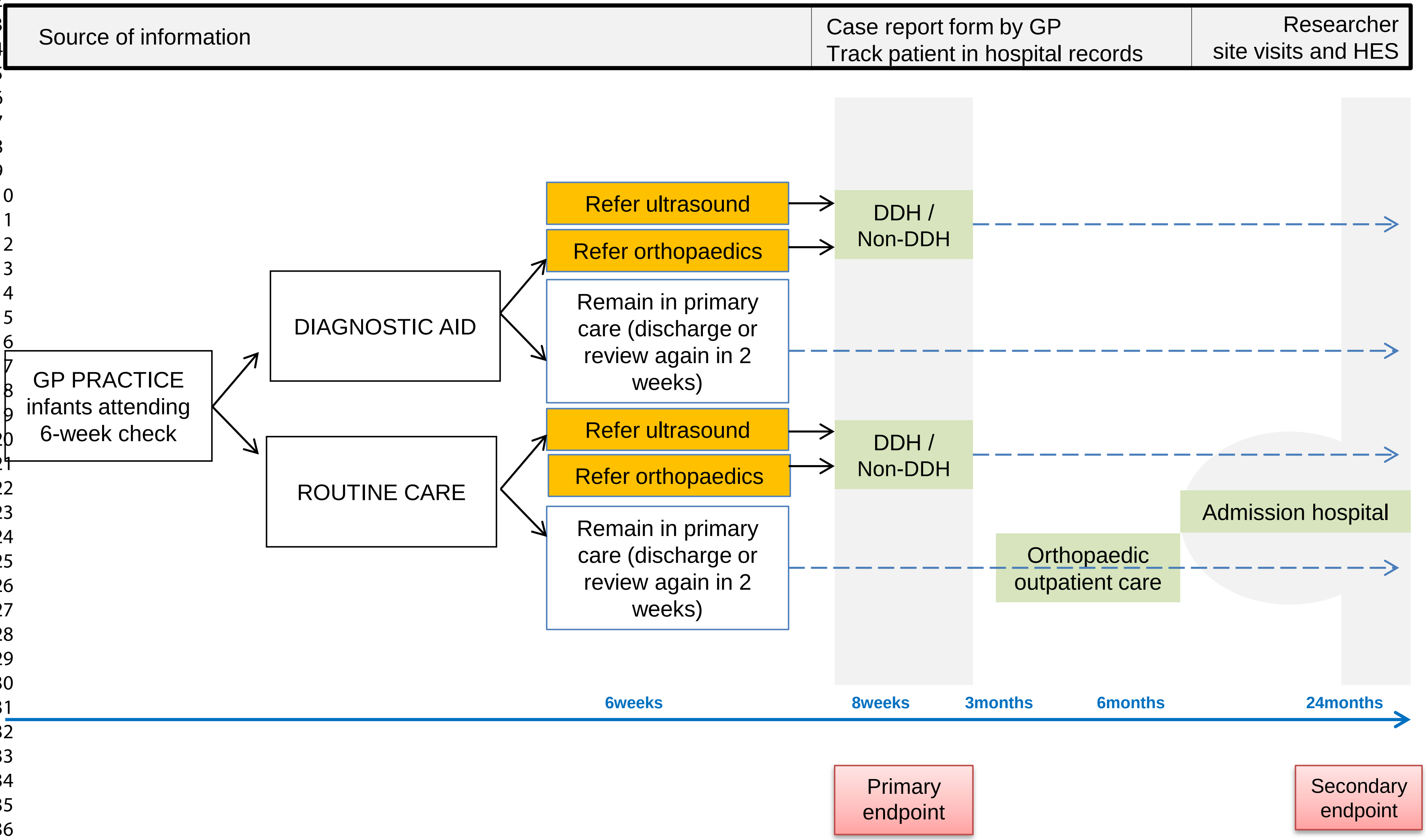
Source of information
Case report form by GP
Track patient in hospital records
Researcher
site visits and HES
GP PRACTICE
infants attending
6-week check
DIAGNOSTIC AID
ROUTINE CARE
Refer ultrasound
Refer orthopaedics
Remain in primary care (discharge or review again in 2 weeks)
Refer ultrasound
Refer orthopaedics
Remain in primary care (discharge or review again in 2 weeks)
DDH /
Non-DDH
DDH /
Non-DDH
Admission hospital
Orthopaedic
outpatient care
6weeks
8weeks
3months
6months
24months
Primary
endpoint
Secondary
endpoint

Appendix 1. ROLES AND RESPONSIBILITIES

Funder

The funding body had or has no involvement in study design; collection, management, analysis and interpretation of data; or the decision to submit for publication. The funding body will be informed of any planned publications, and documentation provided.

Sponsor

The sponsor for this trial is Great Ormond Street Hospital for Children. The sponsor is responsible for providing the investigator with the necessary information to conduct the clinical trial, to ensure proper monitoring of the trial and ensuring compliance to ethical bodies and legislation. The sponsor works to the UK Policy Framework for Health and Social Care Research. The sponsor is not involved in aspects of study design, report writing or data analysis. They are the data controller and all data shall return to the sponsor at the end of the trial. A collaboration agreement is in place with all organisations of the co-investigators. Data processing agreements are in place for situations where data will be collected and processed outside of Great Ormond Street Hospital. The sponsor can be contacted by email (research.governance@gosh.nhs.uk) or telephone 0207 905 2249.

Coordinating centre

PRIMENT Clinical Trials Unit is coordinating this trial. A trial management group has been set up within PRIMENT for the monthly monitoring of the trial conduct. It includes the chief investigator, director of the trials unit, programme manager and trial manager. PRIMENT are responsible for overseeing the conduct and progress of the trial.

Steering committee

The steering committee includes an independent chair, two further independent members (one is a biostatistician), sponsor representative, funder representative, chief investigator and two further members of the research team including. The committee will provide overall

For peer review only

supervision of the trial, review analysis plan, recruitment figures and outcome data, and provide recommendations for any appropriate actions for the trial as necessary.

Data monitoring committee

The steering committee will take on the role of the data monitoring committee.

Data Access

All identifiable data will be stored on encrypted servers, UCL Data Safe Haven. Only restricted members of the research team will be able to access this data. De-identified data will be shared with the wider members of the research team.

For peer review only

Reporting checklist for protocol of a clinical trial.

Based on the SPIRIT guidelines.

Instructions to authors

Complete this checklist by entering the page numbers from your manuscript where readers will find each of the items listed below.

Your article may not currently address all the items on the checklist. Please modify your text to include the missing information. If you are certain that an item does not apply, please write "n/a" and provide a short explanation.

Upload your completed checklist as an extra file when you submit to a journal.

In your methods section, say that you used the SPIRITreporting guidelines, and cite them as:

Chan A-W, Tetzlaff JM, Altman DG, Laupacis A, Gøtzsche PC, Krleža-Jerić K, Hróbjartsson A, Mann H, Dickersin K, Berlin J, Doré C, Parulekar W, Summerskill W, Groves T, Schulz K, Sox H, Rockhold FW, Rennie D, Moher D. SPIRIT 2013 Statement: Defining standard protocol items for clinical trials. Ann Intern Med. 2013;158(3):200-207

		Reporting Item	Page Number
**Administrative information**			
Title	#1	Descriptive title identifying the study design, population, interventions, and, if applicable, trial acronym	1
Trial registration	#2a	Trial identifier and registry name. If not yet registered, name of intended registry	3
Trial registration: data set	#2b	All items from the World Health Organization Trial Registration Data Set	3
Protocol version	#3	Date and version identifier	15
Funding	#4	Sources and types of financial, material, and other support	16
Roles and responsibilities: contributorship	#5a	Names, affiliations, and roles of protocol contributors	1

For peer review only

Roles and responsibilities: sponsor contact information	#5b	Name and contact information for the trial sponsor	Appendix 1
Roles and responsibilities: sponsor and funder	#5c	Role of study sponsor and funders, if any, in study design; collection, management, analysis, and interpretation of data; writing of the report; and the decision to submit the report for publication, including whether they will have ultimate authority over any of these activities	Appendix 1
Roles and responsibilities: committees	#5d	Composition, roles, and responsibilities of the coordinating centre, steering committee, endpoint adjudication committee, data management team, and other individuals or groups overseeing the trial, if applicable (see Item 21a for data monitoring committee)	Appendix 1
**Introduction**			
Background and rationale	#6a	Description of research question and justification for undertaking the trial, including summary of relevant studies (published and unpublished) examining benefits and harms for each intervention	4
Background and rationale: choice of comparators	#6b	Explanation for choice of comparators	5
Objectives	#7	Specific objectives or hypotheses	6
Trial design	#8	Description of trial design including type of trial (eg, parallel group, crossover, factorial, single group), allocation ratio, and framework (eg,	6

For peer review only

		superiority, equivalence, non-inferiority, exploratory)	
**Methods: Participants, interventions, and outcomes**			
Study setting	#9	Description of study settings (eg, community clinic, academic hospital) and list of countries where data will be collected. Reference to where list of study sites can be obtained	7
Eligibility criteria	#10	Inclusion and exclusion criteria for participants. If applicable, eligibility criteria for study centres and individuals who will perform the interventions (eg, surgeons, psychotherapists)	7
Interventions: description	#11a	Interventions for each group with sufficient detail to allow replication, including how and when they will be administered	7
Interventions: modifications	#11b	Criteria for discontinuing or modifying allocated interventions for a given trial participant (eg, drug dose change in response to harms, participant request, or improving / worsening disease)	N/A. The experimental intervention (diagnostic aid) is an electronic tool that cannot be modified. If a practice wishes to withdraw from its use, it can do at any point of the trial. Parent/carers can opt out of its use and any data collection at the 6 week check.
Interventions: adherance	#11c	Strategies to improve adherence to intervention protocols, and any procedures for monitoring adherence (eg, drug tablet return; laboratory tests)	7

For peer review only

Interventions: concomitant care	#11d	Relevant concomitant care and interventions that are permitted or prohibited during the trial	N/A. The experimental intervention (diagnostic aid) is a low risk electronic checklist and does not affect any concomitant care
Outcomes	#12	Primary, secondary, and other outcomes, including the specific measurement variable (eg, systolic blood pressure), analysis metric (eg, change from baseline, final value, time to event), method of aggregation (eg, median, proportion), and time point for each outcome. Explanation of the clinical relevance of chosen efficacy and harm outcomes is strongly recommended	9
Participant timeline	#13	Time schedule of enrolment, interventions (including any run-ins and washouts), assessments, and visits for participants. A schematic diagram is highly recommended (see Figure)	Table 1
Sample size	#14	Estimated number of participants needed to achieve study objectives and how it was determined, including clinical and statistical assumptions supporting any sample size calculations	8
Recruitment	#15	Strategies for achieving adequate participant enrolment to reach target sample size	7
**Methods: Assignment of interventions (for controlled trials)**			

For peer review only

Allocation: sequence generation	#16a	Method of generating the allocation sequence (eg, computer-generated random numbers), and list of any factors for stratification. To reduce predictability of a random sequence, details of any planned restriction (eg, blocking) should be provided in a separate document that is unavailable to those who enrol participants or assign interventions	7
Allocation concealment mechanism	#16b	Mechanism of implementing the allocation sequence (eg, central telephone; sequentially numbered, opaque, sealed envelopes), describing any steps to conceal the sequence until interventions are assigned	7
Allocation: implementation	#16c	Who will generate the allocation sequence, who will enrol participants, and who will assign participants to interventions	7
Blinding (masking)	#17a	Who will be blinded after assignment to interventions (eg, trial participants, care providers, outcome assessors, data analysts), and how	15
Blinding (masking): emergency unblinding	#17b	If blinded, circumstances under which unblinding is permissible, and procedure for revealing a participant’s allocated intervention during the trial	N/A. Trial participants will not be blinded.
**Methods: Data collection, management, and analysis**			
Data collection plan	#18a	Plans for assessment and collection of outcome, baseline, and other trial	9

For peer review only

		data, including any related processes to promote data quality (eg, duplicate measurements, training of assessors) and a description of study instruments (eg, questionnaires, laboratory tests) along with their reliability and validity, if known. Reference to where data collection forms can be found, if not in the protocol	
Data collection plan: retention	#18b	Plans to promote participant retention and complete follow-up, including list of any outcome data to be collected for participants who discontinue or deviate from intervention protocols	7
Data management	#19	Plans for data entry, coding, security, and storage, including any related processes to promote data quality (eg, double data entry; range checks for data values). Reference to where details of data management procedures can be found, if not in the protocol	14
Statistics: outcomes	#20a	Statistical methods for analysing primary and secondary outcomes. Reference to where other details of the statistical analysis plan can be found, if not in the protocol	11
Statistics: additional analyses	#20b	Methods for any additional analyses (eg, subgroup and adjusted analyses)	11
Statistics: analysis population and missing data	#20c	Definition of analysis population relating to protocol non-adherence (eg, as randomised analysis), and any statistical methods to handle missing data (eg, multiple imputation)	12

For peer review only

**Methods: Monitoring**			
Data monitoring: formal committee	#21a	Composition of data monitoring committee (DMC); summary of its role and reporting structure; statement of whether it is independent from the sponsor and competing interests; and reference to where further details about its charter can be found, if not in the protocol. Alternatively, an explanation of why a DMC is not needed	Appendix 1
Data monitoring: interim analysis	#21b	Description of any interim analyses and stopping guidelines, including who will have access to these interim results and make the final decision to terminate the trial	N/A. No interim analysis has been planned for this trial.
Harms	#22	Plans for collecting, assessing, reporting, and managing solicited and spontaneously reported adverse events and other unintended effects of trial interventions or trial conduct	16
Auditing	#23	Frequency and procedures for auditing trial conduct, if any, and whether the process will be independent from investigators and the sponsor	N/A. We have no plans for auditing this trial at the moment. the sponsor may conduct an audit at a random time. The clinical trial unit will conduct monthly central monitoring visits.
**Ethics and dissemination**			
Research ethics approval	#24	Plans for seeking research ethics committee / institutional review board (REC / IRB) approval	15

For peer review only

Protocol amendments	#25	Plans for communicating important protocol modifications (eg, changes to eligibility criteria, outcomes, analyses) to relevant parties (eg, investigators, REC / IRBs, trial participants, trial registries, journals, regulators)	16 and Appendix 1
Consent or assent	#26a	Who will obtain informed consent or assent from potential trial participants or authorised surrogates, and how (see Item 32)	7
Consent or assent: ancillary studies	#26b	Additional consent provisions for collection and use of participant data and biological specimens in ancillary studies, if applicable	N/A. No collection of samples in this trial.
Confidentiality	#27	How personal information about potential and enrolled participants will be collected, shared, and maintained in order to protect confidentiality before, during, and after the trial	9
Declaration of interests	#28	Financial and other competing interests for principal investigators for the overall trial and each study site	16
Data access	#29	Statement of who will have access to the final trial dataset, and disclosure of contractual agreements that limit such access for investigators	Appendix 1
Ancillary and post trial care	#30	Provisions, if any, for ancillary and post-trial care, and for compensation to those who suffer harm from trial participation	N/A. No post trial care is expected. The respective information sheets contain details of Patient Advice Liasion (PALs) services that participants can contact for complaints and compensation.

For peer review only

Dissemination policy: trial results	#31a	Plans for investigators and sponsor to communicate trial results to participants, healthcare professionals, the public, and other relevant groups (eg, via publication, reporting in results databases, or other data sharing arrangements), including any publication restrictions	15
Dissemination policy: authorship	#31b	Authorship eligibility guidelines and any intended use of professional writers	N/A. These have not yet been drawn up.
Dissemination policy: reproducible research	#31c	Plans, if any, for granting public access to the full protocol, participant-level dataset, and statistical code	15
**Appendices**			
Informed consent materials	#32	Model consent form and other related documentation given to participants and authorised surrogates	N/A. This trial is multifaceted and thus has many information sheets and consent forms for different aspects of the trial.
Biological specimens	#33	Plans for collection, laboratory evaluation, and storage of biological specimens for genetic or molecular analysis in the current trial and for future use in ancillary studies, if applicable	N/A. No collection of samples required for this trial.

None The SPIRIT checklist is distributed under the terms of the Creative Commons Attribution License CC-BY-ND 3.0. This checklist can be completed online using https://www.goodreports.org/, a tool made by the EQUATOR Network in collaboration with Penelope.ai

For peer review only

BMJ Open

Study Protocol for evaluation of aid to diagnosis for developmental dysplasia of the hip in general practice: controlled trial randomised by practice

Journal:	*BMJ Open*
Manuscript ID	bmjopen-2020-041837.R1
Article Type:	Protocol
Date Submitted by the Author:	24-Aug-2020
Complete List of Authors:	Roposch, Andreas; UCL, Institute of Child Health; Great Ormond Street Hospital for Children, Department of Orthopaedic Surgery<br>warsame, kaltuun; University College London Institute of Child Health,<br>Chater, Angel; University of Bedfordshire, Department of Sport Science and Physical<br>Green, Judith; Kings College London<br>Hunter, Rachael; University College London, Research Dept of Primary Care and Population Health<br>Wood, J; University College London, PRIMENT Clinical Trials Unit<br>Freemantle, Nick; University College London, Comprehensive Clinical Trials Unit<br>Nazareth, Irwin; UCL, Primary Care and Population Health
**Primary Subject Heading**:	General practice / Family practice
Secondary Subject Heading:	General practice / Family practice
Keywords:	PRIMARY CARE, Paediatric orthopaedics < ORTHOPAEDIC & TRAUMA SURGERY, PAEDIATRICS

SCHOLARONE™
Manuscripts

I, the Submitting Author has the right to grant and does grant on behalf of all authors of the Work (as defined in the below author licence), an exclusive licence and/or a non-exclusive licence for contributions from authors who are: i) UK Crown employees; ii) where BMJ has agreed a CC-BY licence shall apply, and/or iii) in accordance with the terms applicable for US Federal Government officers or employees acting as part of their official duties; on a worldwide, perpetual, irrevocable, royalty-free basis to BMJ Publishing Group Ltd ("BMJ") its licensees and where the relevant Journal is co-owned by BMJ to the co-owners of the Journal, to publish the Work in this journal and any other BMJ products and to exploit all rights, as set out in our licence.

The Submitting Author accepts and understands that any supply made under these terms is made by BMJ to the Submitting Author unless you are acting as an employee on behalf of your employer or a postgraduate student of an affiliated institution which is paying any applicable article publishing charge ("APC") for Open Access articles. Where the Submitting Author wishes to make the Work available on an Open Access basis (and intends to pay the relevant APC), the terms of reuse of such Open Access shall be governed by a Creative Commons licence – details of these licences and which Creative Commons licence will apply to this Work are set out in our licence referred to above.

Other than as permitted in any relevant BMJ Author's Self Archiving Policies, I confirm this Work has not been accepted for publication elsewhere, is not being considered for publication elsewhere and does not duplicate material already published. I confirm all authors consent to publication of this Work and authorise the granting of this licence.

For peer review only

Study Protocol for evaluation of aid to diagnosis for developmental dysplasia of the hip in general practice: controlled trial randomised by practice

Andreas Roposch, Kaltuun Warsame, Angel Chater, Judith Green, Rachael Hunter, John Wood, Nick Freemantle, Irwin Nazareth

Author Affiliations

Andreas Roposch MD MSc FRCS; Kaltuun Warsame BSc

UCL Great Ormond Street Institute of Child Health

30 Guilford Street, London WC1N 3EH

Angel Chater PhD

Faculty of Education and Sport, University of Bedfordshire

Polhill Avenue, Bedford MK41 9EA

Judith Green PhD

Department of Population Health Sciences, Kings College London

Great Maze Pond, London SE1 1UL

Nick Freemantle PhD

Institute of Clinical Trials and Methodology, University College London

90 High Holborn, London WC1V 6LJ

Irwin Nazareth PhD FRCGP; Rachael Hunter MSc; John Wood MSc

Research Department for Primary Care and Population Health, University College London

Rowland Hill Street, London NW3 2PF

Corresponding author: Professor Andreas Roposch, UCL Great Ormond Street Institute of Child Health, 30 Guilford Street, London WC1N 3EH - a.roposch@ucl.ac.uk

Keywords: Developmental dysplasia of the hip, primary care, diagnostic aid

For peer review only

ABSTRACT

Introduction: In the UK, a compulsory '6-week hip check' is performed in primary care for the detection of developmental dysplasia of the hip (DDH). However, missed diagnoses and infants incorrectly labelled with DDH remain a problem, potentially leading to adverse consequences for infants, their families and the NHS. National policy states that infants should be referred to hospital if the 6-week check suggests DDH, though there is no available tool to aid examination or offer guidelines for referral. We developed standardised diagnostic criteria for DDH, based on international Delphi consensus, and a 9-item checklist that has the potential to enable non-experts to diagnose DDH in a manner approaching that of experts.

Methods and Analysis*:* We will conduct a controlled trial randomised by practice that will compare a diagnostic aid against standard care for the hip check. The primary objective is to determine whether an aid to the diagnosis of DDH reduces the number of clinically insignificant referrals from primary care to hospital, and the number of late diagnosed DDH. The trial will include a qualitative process evaluation, an assessment of professional behaviour change and a full health economic evaluation. We will recruit 152 GP practices in England. These will be randomised to conduct the hip checks with use of the study diagnostic aid and/or as per usual practice. The total number of infants seen during a 15-month recruitment period will be 110 per practice. Two years after the 6-week hip check we will measure the number of referred infants that are (1) clinically insignificant for DDH and (2) those that constitute appropriate referrals.

Ethics and Dissemination: This study has approval from the Health Research Authority and the Confidentiality Advisory Group. Results will be published in peer-reviewed academic journals, disseminated to patient organisations and the media.

Registration details: clinicaltrials.gov (NCT04101903; Pre-results).

For peer review only - http://bmjopen.bmj.com/site/about/guidelines.xhtml

STRENGTHS AND LIMITATIONS OF THIS STUDY

- This is the first trial to evaluate a diagnostic aid for the 6-week check with reference to evaluating both missed and unnecessary referrals to hospital.
- Implementable on existing clinical software used by GPs, the proposed aid will be easy to use.
- A comprehensive process evaluation, using qualitative methods and behaviour change frameworks, will be done alongside to the trial; plus a full health economic evaluation.
- The reliance on existing practice staff to report monthly updates is essential but could pose a risk to timely and complete data collection.
- The collection of identifiable data through site visits across 152 practices will be challenging.

For peer review only

INTRODUCTION

Developmental dysplasia of the hip (DDH) is characterized by varying displacement of the proximal femur from the acetabulum with associated acetabular dysplasia. Dislocation occurs in 1-2/1000 infants per year but milder forms occur in 40-60/1000.[1] Early recognition of disease is associated with better outcomes. It is national policy[2] to examine all infants for the presence of DDH at birth and at age 6-8 weeks in primary care (6-week hip check). If diagnosed within the first 6-8 weeks, splinting of the hips is successful in 85% of cases.[3] Later diagnoses usually will require invasive treatment, with many years of continued monitoring.[4] Late diagnosed DDH is a common cause of medical negligence claims, with increased suffering for affected patients.

Timely diagnoses of DDH remain a challenge despite the compulsory 6-week check.[5] In one study, the median age at diagnosis was 14 months, with only 40% of infants diagnosed during routine examinations and 60% presenting owing to parental concerns.[6] In another study, 30% of infants were not diagnosed by 12 weeks.[7] Because there is no further compulsory check after that at 6-weeks, it is vital that the 6-week check is effective. Also infants incorrectly identified with "DDH" in primary care and referred to hospital remain a challenge for both families and NHS. This group does not

For peer review only - http://bmjopen.bmj.com/site/about/guidelines.xhtml

require treatment and reassurance provided in primary care would avoid unnecessary anxiety and inefficient use of hospital resources. Of 1,918 infants referred to hospital from primary care for DDH, only 64 (3%) had DDH[7] but 1,270 (66%) were identified as 'DDH' based on inappropriate criteria,[8] e.g. "crease asymmetry" (n=234) or "click" (n=648). If GPs were able to discriminate better between benign abnormalities requiring reassurance (perhaps with a follow-up in primary care) and findings requiring referrals, outcomes of infants with *and without* DDH would improve.

In prior research we suggested that too many diagnostic criteria[8] and variability in the use of diagnostic criteria among clinicians[9] complicate the task of diagnosing DDH. We developed standardised diagnostic criteria to reduce the variability in assessment and management decisions in infants examined for DDH.[10] The weighted criteria demonstrated validity.[10] In a study of 44 patients referred from GPs to hospital, the weighted criteria demonstrated a positive predictive value of 89% (95% confidence interval 70, 97) and negative predictive value of 76% (95% confidence interval, 50-96).[11] We refined these binary criteria, in form of a checklist with 9 items, for use by GPs during the 6-week hip check and developed a training video, featuring a GP. This checklist and video were refined in a feasibility study which used qualitative methods to explore the acceptability of the format, style and delivery of the intervention (report available on request). The video explains the meaning of the diagnostic criteria; it also demonstrates how precisely to elicit the diagnostic criteria. For example, the video explains the difference between the Barlow and Ortolani manoeuvres, how to test for a leg length inequality, or how to identify limitations in hip abduction.

Rationale

Current referral patterns suggest there could be considerable health gains from improved diagnostic and referral decisions at the 6-week check.[5] GPs, in a preparatory focus group, identified the need for a 'comprehensive, structured guide' for the 6-week check. Diagnostic aids enable physicians to overcome barriers in diagnostic reasoning[12] by shifting intuitive to analytical aspects of diagnostic reasoning. Decisions made under these circumstances approach normative reasoning and rationality

For peer review only - http://bmjopen.bmj.com/site/about/guidelines.xhtml

more closely, and are more reliable and safer.[13] Building on our earlier research, we propose to facilitate GP's diagnostic reasoning at the 6-week check by structuring current practice with use of a previously developed diagnostic aid. A diagnostic aid of this kind provides a structured approach to the assessment of infants and offers guidance about referrals.

Objectives

- To determine whether an aid to the diagnosis of DDH reduces the number of clinically insignificant referrals from primary care to hospital, and the number of late diagnosed DDH.
- To determine the cost-effectiveness of this intervention.
- To conduct an integrated qualitative and quantitative process evaluation in order to understand all participants' experience in the trial and with the intervention; study how the intervention is implemented; investigate contextual factors that affect the intervention.

METHODS AND ANALYSIS

The SPIRIT reporting guidelines were used in the preparation of this clinical trial protocol.[14]

Design

This is a cluster randomised controlled trial of GP practices in England, with randomisation and intervention at practice level but the primary endpoint measured at patient level (Figure 1). An internal pilot will be done in the first 4 months to ascertain accrual rates – we will progress to the main trial if we succeed in recruiting ≥12 practices per month (expected recruitment is ≥21 practices/month for the main trial). Incorporated in the trial are (i) a process evaluation investigating determinants of GPs' referral behaviour and the implementation of the intervention in practice; and (ii) a health economic evaluation in a child's lifetime.

Eligibility and recruitment

For peer review only - http://bmjopen.bmj.com/site/about/guidelines.xhtml

GP practices registered in England that carry out 6-week hip checks (at age 42-76 days) and agree (i) to being randomised and (ii) to hospitals releasing data concerning infants they examine in the trial. Ineligible are practices planning to close with 12 months of trial start.

We anticipate that ≥110 infants per year will undergo the 6-week hip check in each of the recruited practices. Each practice will recruit infants for a period of 15 months and infants will be followed for 2 years. This trial is planned therefore to run for 44 months across 152 GP practices.

Retention

The proposed trial is not onerous on the practice staff and practices will not be challenged by excessive study-related manoeuvres. Because the primary outcome will be collected using practice and hospital databases only, it is likely that we will attain high levels of follow up. We will send periodic newsletters to GPs and foster regular contacts.

Randomisation

Practices will submit an eligibility form to the clinical trials unit, which will register and randomly allocate (concealed) practices within 48 hours. An independent statistician will coordinate randomisation with an allocation ratio of 1:1 and stratified by practice size (based on observed sizes obtained from expressions of interest – we expect 2 or 3 strata).

Interventions

Control intervention: GPs will assess infant hips following best practice principles; they will be provide a leaflet about the national best practice recommendations.[2]

Experimental intervention: This is a complex intervention comprising a video developed specifically for this trial and a diagnostic aid in form of a 9-item binary checklist. At the time when informed consent is taken, GPs will watch the video. They will then examine all infants in the 6-week check according to best practice principles, but with the addition of the diagnostic aid (implemented electronically to each practice's computer system).

For peer review only

For peer review only - http://bmjopen.bmj.com/site/about/guidelines.xhtml

According to national guidelines, the 6-week check shall capture infants at age 6-10 weeks and infants in whom a diagnosis of DDH is considered should see a specialist within 2 weeks.[2] Participating GPs of both intervention arms will be asked to adhere to this policy.

Definitions for the purpose of this trial

'Appropriate referral' denotes a referred hip deemed 'clinically significant', i.e. is treated or monitored (at least one follow-up) by a specialist surgeon. Any ambiguous cases will be reviewed by our Expert Advisory Panel who will assign an ultimate diagnosis in consensus. 'Clinically insignificant' are referred hips resulting in reassurance and discharge from surgeons' clinics (i.e. no treatment or monitoring). 'Late diagnoses' are cases of DDH diagnosed by a specialist surgeon at age 3-24 months.[15;16]

Sample size calculation

We based its calculation on Poisson regression and audit data from the Nottingham area (partially presented in Price et al[7]) and University College Hospital (audit of 3 years' practice, unpublished data): a conservative estimate of the number of infants referred as a consequence of the 6-week check within the recruitment period of one year is, on average, 3 per practice. Based on the same data, we estimate that about 1 in 3 (or, on average, 1 per practice) of such referrals is correctly made ('appropriate' referrals, defined above as 'clinically significant', are a subset of these 'correct' referrals). Thus, on average, we expect 2 'incorrect' referrals per practice. These are the ones we seek to reduce, and the intervention should serve to cut them by 50%. Thus we aim to detect a reduction, on average, from 3 to 2 referrals per practice in the intervention arm. To account for correlation within practices, which can also be thought of as overdispersion relative to the underlying Poisson variation, we assumed a between-practice component of variance of 20% of the average Poisson 'counting' variance per practice. For 90% statistical power and testing 2-sided at p=0.05, we need 76 practices per group (15% of this total comprises a safety margin to allow for potential challenges). Average referral rates from the Nottingham (2.8%) and University College Hospital data

For peer review only - http://bmjopen.bmj.com/site/about/guidelines.xhtml

(2.7%) are very consistent, and suggest a target of about 110 children per practice per year to undergo the 6-week check.

Primary trial endpoint

Number of referred infants that are considered 'clinically insignificant' – this is a measure of the clinical importance of referrals in the 2-week hip pathway. We chose this outcome because it is extremely relevant clinically and one for which it is possible to power the trial.[2] An intervention could be successful at achieving this outcome whilst missing infants who do have DDH; we thus specified a *principal secondary endpoint*. False negatives are also important but, because of their rarity, would not be an efficient endpoint on which to power the trial (and would thus be outside ICH E9,[17] which recommends that the primary outcome should be both clinically and statistically convincing). A research assistant will retrieve these data from respective hospital electronic systems by conducting site visits to all secondary care facilities to which GPs refer infants, starting 6 weeks after the last patient enters the trial.

Secondary trial endpoints (Table 1)

Infant level

• Number of appropriate referrals per practice (principal secondary endpoint): for all infants referred as a consequence of the 6-week check, a researcher will collect, using unique NHS patient numbers, and categorise the appropriateness of referrals by a practice with respective hospital databases using a standardised taxonomy, blind to practice random allocation. S/he will conduct site visits to all such hospitals starting 6 weeks after the last patient entered the trial.

• Number of late diagnoses: We will employ deterministic methods of data linkage. Using unique NHS numbers of any recruited infant, we will obtain from Health & Social Care Information Centre the corresponding Health Episodes Statistic (HES) identifiers. HES is a data warehouse that includes all hospital admissions and outpatients' visits occurring in all hospitals in England. In HES we will establish whether any infant in the trial was admitted to a UK hospital as a result of DDH using

For peer review only - http://bmjopen.bmj.com/site/about/guidelines.xhtml

relevant ICD-10 codes and OPCS-4 codes.[18] We will extract, for the whole trial period, the full hospital history for all infants in the trial (in and out patient episodes), collate all episodes into a combined exploratory analysis view, collate interactions, and allocate outcomes to practice groups to facilitate outcome analyses.[19] These data will be compared with the data collected by the researcher (see above); the use of these 2 strategies will enhance the robustness of data.

• Consequences of late diagnoses: Using data collected above we will record nature and frequency of such consequences (nature of treatment, length of hospital stay, frequency of secondary care contacts).

• Health-related quality of life (parental proxy report): Child-Health-Utility-9D.[20]

Process level

• Volume of referrals: Total number of patients referred to secondary care during the trial period. This variable will be collected prospectively at practice level by the participating GP and forwarded monthly to the clinical trial unit.

• Timeliness of referrals: In infants referred to secondary care, we will measure the days from referral issued to hospital appointment (collected in the same way as primary endpoint). This process measure will inform about target wait times[2] achieved.

Clinician level

• Confidence and attitudes of GP and secondary care clinicians towards the diagnostic aid: will be assessed 12 weeks after trial set-up and at trial completion using a modified measure based on the Theory of Planned Behaviour.[21]

• Implementation issues and acceptability of diagnostic aid among GPs and secondary care providers: we will conduct qualitative research at the trial end to elicit this information.

• Use of diagnostic aid and acceptability of intervention: a self-administered questionnaire to evaluate the use of the aid will be posted to all the GPs in the intervention group; also direct observations and interviews will ascertain this outcome.

For peer review only - http://bmjopen.bmj.com/site/about/guidelines.xhtml

For peer review only

Parent/carer level (collected from one in ten parents/carers)

• General worry: State-Trait Anxiety Inventory 6-items short form.[22]

• DDH-related worry: Infant Hip Worries Inventory.[23]

• Satisfaction with trial: dimensions of care items from EUROPEP.[24]

For peer review only

For peer review only - http://bmjopen.bmj.com/site/about/guidelines.xhtml

Table 1. Assessments at different time points

	**Baseline**	**Within 2 weeks of consent**	**At 6-week hip check**	**3-month follow-up**	**15-month follow-up**	**2-year follow-up**
Clinician-level						
Theoretical Domains Framework questionnaire	X		X	X	X	X
GP characteristics questionnaire	X					
Fidelity questionnaire			X	X	X	X
Non-participant observations			X			
Semi-structured interviews			X		X	
Parent/carer-level						
State Trait Anxiety Inventory 6 item short form			X			
Infant Hip Worries Inventory			X			
EUROPEP			X			
Non-participant observations			X			
Semi-structured interviews		X				
Out-of-pocket-costs questionnaire			X			
EuroQol 5 Dimension Scale (EQ-5D)			X			X
Child Health Utility 9D (CHU 9D)						X
Willingness-to-pay questionnaire					X	

For peer review only

Statistical analysis

Primary endpoint: We will compare the randomised practices using Poisson mixed models, accounting for extra *Poissonian* variability by including random intercept terms for practices. The response variable will be the number of clinically insignificant referrals from each practice with an offset in the linear model of the log(e) total number of children checked in each practice, to account for differences in practice size and constitution. The random effect at the practice level will account for overdispersion.

Principal secondary endpoint: This will be analysed as above; however, counts here are expected to be lower. Should they be insufficient to support the full model, the model will be simplified by (e.g. use of additive variance component term rather than a generalised random intercept term to address overdispersion). We will describe the planned statistical analysis and its adaptation where there are difficulties in achieving convergence in a pre-specified statistical analysis plan.

Late diagnoses: As we anticipate small numbers of events in each randomised comparison we may not be in a position to account for practices using random intercept terms. Where this is the case we will report the total numbers over a minimum 2-year observation and compare the overall group scores using Fishers exact test.

Quantitative outcome measures: We will summarize scores by instrument, accounting for practices and report difference in group means for each treatment arm. We expect mean scores to be lower in the intervention arm for State-Trait Anxiety Inventory and Infant Hip Worries Inventory, but higher or equal for EUROPEP.

Missing data: For the primary endpoint and principal secondary endpoint the data collection methods should identify qualifying episodes. Because of the nature of these data the conventional concept of missingness does not directly apply (eg we will not have individually randomised subjects who cannot be followed up). However if a practice withdraws from the trial we will explore the consequences of this action by assuming a poor outcome among that practice if in the intervention

For peer review only

group and a good outcome if in the control condition, to identify the potential consequences of their withdrawal. Complete case analyses will be conducted for secondary outcomes. If there is a mismatch between practices in the two treatment conditions we will consider undertaking joint models examining simultaneously the binomial of missingness and the outcome measure of interest.

Health economics

Cost analysis

We will analyse the cost associated with the intervention compared with usual practice for the entire trial period, and examine costs from the perspective of the NHS and of families. The cost components included in main analysis are: cost of 6-week in both intervention arms; any subsequent referrals, diagnostic tests and treatment. We will collect costs about GP time, which we will multiply by unit costs from routine sources.[25]

Within-trial cost-effectiveness analysis

With the costs described above we will produce a dataset of patient-level within-trial costs and outcomes. We will calculate the incremental cost per clinically insignificant referral avoided and the incremental cost per late diagnosis avoided. Using bootstrapping of the mean cost and outcomes differences, we will estimate confidence intervals around the incremental cost-effectiveness ratios.[26] With the bootstrap replications we will construct a cost-effectiveness acceptability curve to show the probability that the aid is cost-effective for different values of NHS willingness to pay for outcomes. We will perform deterministic sensitivity analyses.

Long-run cost utility analysis

We will use several measures to evaluate the lifetime cost-effectiveness of the intervention. We will ask 20 carers of infants aged 2-4 years to complete (1) on behalf of their children the Child-Health-utility-9D[20] and (2) for themselves the EQ-5D-5L,[27] both measure health-related quality of life.

Cost-benefit analysis

For peer review only - http://bmjopen.bmj.com/site/about/guidelines.xhtml

With data from the trial about the impact of the intervention on appropriate referrals, we will calculate the monetary value that parents place on the intervention using willingness-to-pay methodology.[28] This will provide an estimate of the monetary value of the additional benefits (positive/negative) of the intervention. We will calculate the net benefit of the intervention by subtracting the incremental cost of the aid, as calculated above from the trial data, from the monetary value of the additional benefit. Following recruitment of the last infant in the trial, we will recruit 200 carers of infants undergoing the 6-week check from trial-participating practices. These will be 100 carers whose infants will be referred to secondary care as a consequence of the 6-week check and 100 who will not. They will complete a self-report questionnaire that utilised several techniques[28] to elicit willingness-to-pay values. We will calculate willingness-to-pay values for the whole sample and test for variations based on socio-demographic groups and referral to secondary care.

Integrated qualitative and quantitative process evaluation

This workstream will explore the implementation, adherence to protocol, receipt and setting of the intervention. We will examine the views of all groups of participants on the intervention; study how the intervention is implemented; investigate contextual factors that affect the intervention; and study how effects vary in subgroups of GPs. These data will help in understanding how, for whom and why the trial had effects and the extent to which outcomes result from issues of trial fidelity and implementation. We will collect process data from all 152 sites including clinician and carer outcomes. We will conduct alongside the trial non-participant observation and semi-structured interviews. We will include a purposive sample of 10 practices for the qualitative study, interviewing 4-5 participants in each (e.g. GP, carers, hospital consultant), resulting in 40-50 interviews. This sample will include a small number of practices in the control arm (for comparative purposes), and a range of intervention practices to include different locations, practice sizes and types. We will analyse process data before outcome data to avoid bias in interpretation.[29] Interviews will be audio-

For peer review only - http://bmjopen.bmj.com/site/about/guidelines.xhtml

For peer review only

recorded and transcribed, data from observations will be recorded contemporaneously using a template. Data from process outcomes, interviews (transcripts) and observations will be analysed from the perspective of both behaviour change theory[30] and normalization process theory.[31]

Strategies to mitigate potential bias

Since this effectiveness trial will test whether the intervention can work under usual circumstances, we will rely on paediatric orthopaedic surgeons in determining the ultimate diagnosis of DDH. Variations in the surgeons' diagnostic accuracy are inevitable hence the need for a randomised study. We will perform analyses by surgeon (or hospital) to quantify this variation. Blinding of GPs, practice staff, carers is impossible; however, most such outcomes will be assessed with validated questionnaires. Primary and principal secondary endpoints will be collected by an independent researcher blinded to treatment allocation. In case an infant is referred to hip ultrasound without an orthopaedic consultation, a trial-appointed advisory panel shall review the scan blinded and according to standard methods[32] to avoid reporting bias. There is a risk for verification bias – while our trial includes a 2-year follow up to capture late presenting DDH, we cannot rule out that some infants with DDH will remain undiagnosed within this period, thus underestimating the number of late diagnosed DDH. However, the 2-year mark has previously been found to be a robust outcome.[33]

Patient and public involvement

We developed this protocol with carers of children with DDH and the founding director of 'Steps', a charity supporting patients with lower limb disorders. We discussed the need for the trial and trial procedures and conduct with staff members of GP practices. Our established patient and public involvement group has reviewed and commented on this protocol and will periodically review, support and advise on the conduct of the trial.

Trial and data management

The trial will be run through PRIMENT Clinical Trials Unit and conducted in accordance with established quality management systems and standardised operating procedures (Appendix 1). All

For peer review only - http://bmjopen.bmj.com/site/about/guidelines.xhtml

data will be handled in accordance with the UK Data Protection Act 2018. All analyses will be conducted blinded to allocation groups.

Ethics and dissemination

Leicester Central Research Ethics Committee (19/EM/0317) and Health Research Authority Confidentiality Advisory Group (19/CAG/0198) approved protocol version 2.0 (12/11/2020). The latter granted ‘Section 251’ approval as operating a consented model was unfeasible within the clinical setting (written informed consent will be obtained at cluster level from lead GPs). It is registered at clinicaltrials.gov (NCT04101903). The results will be published in peer-reviewed academic journals, disseminated to patient organisations and the media.

Discussion

This trial is part of a programme of research to improve the diagnosis of DDH: consensus-based diagnostic criteria were established in prior research, tailored for use in primary care, supplemented by a video designed for GPs. There has only been one randomised trial on the topic of DDH in the UK[33] but it explored the use of ultrasound screening – our trial focusses on the compulsory ‘6-week check’. Because the intervention tested in this trial is based on consensus of clinical experts, there is a risk that the opinions of experts change as clinical knowledge evolves. However, the criteria of the diagnostic aid have been in use for decades and will likely not loose relevance in the foreseeable future. The collection of outcome data from various hospitals connected to GP practices will be challenging; use of national health services databases should mitigate this challenge. While our trial includes a 2-year followup period to capture late presenting DDH, we cannot rule out that some infants with DDH will remain undiagnosed within this period. This trial has the potential to improve the compulsory 6-week hip check with use of a relatively simple intervention. It will also provide an understanding of the cost effectiveness of the intervention in a whole lifetime horizon of a 6-week old. If successful, the intervention can be rolled out to clinical services relatively easily and at low costs.

For peer review only - http://bmjopen.bmj.com/site/about/guidelines.xhtml

Author contributions

AR, IN and NF were involved in conception and trial design. JG expanded and developed the qualitative process evaluation of the protocol. AC designed the behaviour change psychology aspects of the trial, working closely with JG and AR. RH developed the health economic evaluation plan, with input from AR and IN. JW and IN developed all statistical aspects of the trial, with input from AR and IN. AR and KW drafted the manuscript for publication. IN, JG, AC, IN, JW, NF, RH, KW and AR critically revised the article for important intellectual content and approved the final version of the manuscript. AR is chief investigator of the trial and together with IN, NF, JW, JG, AC secured its funding. We acknowledge the valuable input from Professor Daniel Ray in relation to the collaborative work with NHS Digital for outcome data collection.

Funding statement

This paper presents independent research funded by the National Institute for Health Research (NIHR) under its Programme Grant for Applied Research funding stream (RP-PG-0616-20006). The views expressed are those of the authors and not necessarily those of the NIHR or the Department of Health and Social Care.

Competing interest statement

None

References

(1) Bialik V, Bialik GM, Blazer S, Sujov P, Wiener F, Berant M. Developmental dysplasia of the hip: a new approach to incidence. *Pediatrics* 1999; 103(1):93-99.

(2) Public Health England. Newborn and infant physical examination (NIPE) sceening handbook. www.gov.uk/government/publications/newborn-and-infant-physical-examination-programme-handbook/newborn-and-infant-physical-examination-screening-programme-handbook . 27-8-2019.

For peer review only

For peer review only - http://bmjopen.bmj.com/site/about/guidelines.xhtml

(3) Clarke NM, Reading IC, Corbin C, Taylor CC, Bochmann T. Twenty years experience of selective secondary ultrasound screening for congenital dislocation of the hip. *Arch Dis Child* 2012; 97(5):423-429.

(4) NJR Editorial Board. 10th Annual report: national joint registry for England, Wales and Northern Ireland. 2013.

(5) Davies R, Talbot C, Paton R. Evaluation of primary care 6- to 8-week hip check for diagnosis of developmental dysplasia of the hip: a 15-year observational cohort study. *Br J Gen Pract* 2020; 70(693):e230-e235.

(6) Sanghrajka AP, Murnaghan CF, Shekkeris A, Eastwood DM. Open reduction for developmental dysplasia of the hip: failures of screening or failures of treatment? *Ann R Coll Surg Engl* 2013; 95(2):113-117.

(7) Price KR, Dove R, Hunter JB. Current screening recommendations for developmental dysplasia of the hip may lead to an increase in open reduction. *Bone Joint J* 2013; 95-B(6):846-850.

(8) Roposch A, Liu LQ, Hefti F, Clarke NM, Wedge JH. Standardized diagnostic criteria for developmental dysplasia of the hip in early infancy. *Clin Orthop Relat Res* 2011; 469(12):3451-3461.

(9) Roposch A, Liu LQ, Protopapa E. Variations in the use of diagnostic criteria for developmental dysplasia of the hip. *Clin Orthop Relat Res* 2013; 471(6):1946-1954.

(10) Roposch A, Protopapa E, Cortina-Borja M. Weighted diagnostic criteria for developmental dysplasia of the hip. *J Pediatrics* 2014; 165:1236-1240.

(11) Roposch A, Protopapa E. Testing newly developed standardized diagnostic criteria for DDH in consecutive patients referred to a paediatric orthopaedic unit. *Pediatric Orthopedic Society of North America* 2013.

(12) El-Kareh R, Hasan O, Schiff GD. Use of health information technology to reduce diagnostic errors. *BMJ Qual Saf* 2013; 22 Suppl 2:ii40-ii51.

(13) Croskerry P. Clinical cognition and diagnostic error: applications of a dual process model of reasoning. *Adv Health Sci Educ Theory Pract* 2009; 14 Suppl 1:27-35.

(14) Chan AW, Tetzlaff JM, Altman DG, Laupacis A, Gotzsche PC, Krleza-Jeric K et al. SPIRIT 2013 statement: defining standard protocol items for clinical trials. *Ann Intern Med* 2013; 158(3):200-207.

(15) Woolacott NF, Puhan MA, Steurer J, Kleijnen J. Ultrasonography in screening for developmental dysplasia of the hip in newborns: systematic review. *BMJ* 2005; 330(7505):1413.

(16) Clinical practice guideline: early detection of developmental dysplasia of the hip. Committee on Quality Improvement, Subcommittee on Developmental Dysplasia of the Hip. American Academy of Pediatrics. *Pediatrics* 2000; 105(4 Pt 1):896-905.

For peer review only - http://bmjopen.bmj.com/site/about/guidelines.xhtml

(17) ICH Steering Committee. ICH harmonised tripartite guideline: Statistical principles for clinical trials. https://database.ich.org/sites/default/files/E9_Guideline . 5-2-1998.

(18) NHS Digital. National clinical coding standards OPCS-2 (2018). Norwich, England: TSO Shop; 2018.

(19) MacArthur C, Jolly K, Ingram L, Freemantle N, Dennis CL, Hamburger R et al. Antenatal peer support workers and initiation of breast feeding: cluster randomised controlled trial. *BMJ* 2009; 338:b131.

(20) Stevens K. Assessing the performance of a new generic measure of health-related quality of life for children and refining it for use in health state valuation. *Appl Health Econ Health Policy* 2011; 9(3):157-169.

(21) Powell AA, Bloomfield HE, Burgess DJ, Wilt TJ, Partin MR. A conceptual framework for understanding and reducing overuse by primary care providers. *Med Care Res Rev* 2013; 70(5):451-472.

(22) Marteau TM, Bekker H. The development of a six-item short-form of the state scale of the Spielberger State-Trait Anxiety Inventory (STAI). *Br J Clin Psychol* 1992; 31 (Pt 3):301-306.

(23) Gardner F, Dezateux C, Elbourne D, Gray A, King A, Quinn A et al. The hip trial: psychosocial consequences for mothers of using ultrasound to manage infants with developmental hip dysplasia. *Arch Dis Child Fetal Neonatal Ed* 2005; 90(1):F17-F24.

(24) Wensing M. EUROPEP 2006: Revised EUROPEP instrument and user manual. www.equip.ch/files/25/europep_2006rapport.pdf 6-6-2014.

(25) Department of Health. Department of Health. National Schedule of Reference Costs - Year 2011-12 - NHS Trusts and NHS Foundation Trusts: NHS own costs. 2015.

(26) Briggs AH, Wonderling DE, Mooney CZ. Pulling cost-effectiveness analysis up by its bootstraps: a non-parametric approach to confidence interval estimation. *Health Econ* 1997; 6(4):327-340.

(27) Herdman M, Gudex C, Lloyd A, Janssen M, Kind P, Parkin D et al. Development and preliminary testing of the new five-level version of EQ-5D (EQ-5D-5L). *Qual Life Res* 2011; 20(10):1727-1736.

(28) McIntosh E. Using discrete choice experiments within a cost-benefit analysis framework: some considerations. *Pharmacoeconomics* 2006; 24(9):855-868.

(29) Oakley A, Strange V, Bonell C, Allen E, Stephenson J. Process evaluation in randomised controlled trials of complex interventions. *BMJ* 2006; 332(7538):413-416.

(30) Michie S, Richardson M, Johnston M, Abraham C, Francis J, Hardeman W et al. The behavior change technique taxonomy (v1) of 93 hierarchically clustered techniques: building an international consensus for the reporting of behavior change interventions. *Ann Behav Med* 2013; 46(1):81-95.

For peer review only - http://bmjopen.bmj.com/site/about/guidelines.xhtml

(31) Murray E, Treweek S, Pope C, MacFarlane A, Ballini L, Dowrick C et al. Normalisation process theory: a framework for developing, evaluating and implementing complex interventions. *BMC Med* 2010; 8:63.

(32) Roposch A, Moreau NM, Uleryk E, Doria AS. Developmental dysplasia of the hip: quality of reporting of diagnostic accuracy for US. *Radiology* 2006; 241(3):854-860.

(33) Elbourne D, Dezateux C, Arthur R, Clarke NMP, Gray A, King A et al. Ultrasonography in the diagnosis and management of developmental hip dysplasia (UK Hip Trial): clinical and economic results of a multicentre randomised controlled trial. *Lancet* 2002; 360(9350):2009-2017.

Figure legends

Figure 1. Flowchart demonstrating pathways and principal endpoints and their collection.

For peer review only

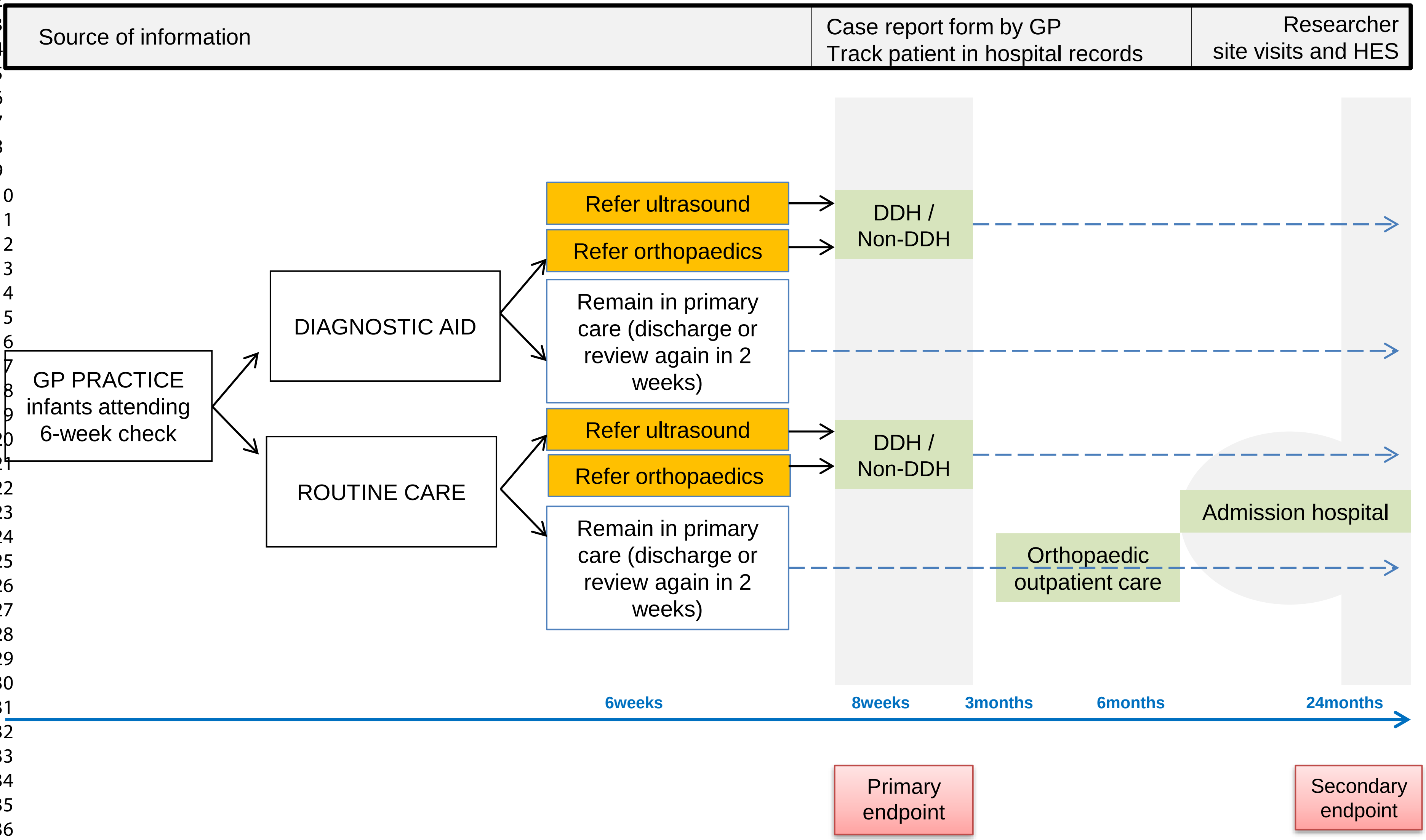
Source of information
Case report form by GP
Track patient in hospital records
Researcher
site visits and HES
GP PRACTICE
infants attending
6-week check
DIAGNOSTIC AID
ROUTINE CARE
Refer ultrasound
Refer orthopaedics
Remain in primary care (discharge or review again in 2 weeks)
Refer ultrasound
Refer orthopaedics
Remain in primary care (discharge or review again in 2 weeks)
DDH / Non-DDH
DDH / Non-DDH
Admission hospital
Orthopaedic outpatient care
6weeks
8weeks
3months
6months
24months
Primary endpoint
Secondary endpoint

Appendix 1. ROLES AND RESPONSIBILITIES

Funder

The funding body had or has no involvement in study design; collection, management, analysis and interpretation of data; or the decision to submit for publication. The funding body will be informed of any planned publications, and documentation provided.

Sponsor

The sponsor for this trial is Great Ormond Street Hospital for Children. The sponsor is responsible for providing the investigator with the necessary information to conduct the clinical trial, to ensure proper monitoring of the trial and ensuring compliance to ethical bodies and legislation. The sponsor works to the UK Policy Framework for Health and Social Care Research. The sponsor is not involved in aspects of study design, report writing or data analysis. They are the data controller and all data shall return to the sponsor at the end of the trial. A collaboration agreement is in place with all organisations of the co-investigators. Data processing agreements are in place for situations where data will be collected and processed outside of Great Ormond Street Hospital. The sponsor can be contacted by email (research.governance@gosh.nhs.uk) or telephone 0207 905 2249.

Coordinating centre

PRIMENT Clinical Trials Unit is coordinating this trial. A trial management group has been set up within PRIMENT for the monthly monitoring of the trial conduct. It includes the chief investigator, director of the trials unit, programme manager and trial manager. PRIMENT are responsible for overseeing the conduct and progress of the trial.

Steering committee

The steering committee includes an independent chair, two further independent members (one is a biostatistician), sponsor representative, funder representative, chief investigator and two further members of the research team including. The committee will provide overall

For peer review only

supervision of the trial, review analysis plan, recruitment figures and outcome data, and provide recommendations for any appropriate actions for the trial as necessary.

Data monitoring committee

The steering committee will take on the role of the data monitoring committee.

Data Access

All identifiable data will be stored on encrypted servers, UCL Data Safe Haven. Only restricted members of the research team will be able to access this data. De-identified data will be shared with the wider members of the research team.

For peer review only

Reporting checklist for protocol of a clinical trial.

Based on the SPIRIT guidelines.

Instructions to authors

Complete this checklist by entering the page numbers from your manuscript where readers will find each of the items listed below.

Your article may not currently address all the items on the checklist. Please modify your text to include the missing information. If you are certain that an item does not apply, please write "n/a" and provide a short explanation.

Upload your completed checklist as an extra file when you submit to a journal.

In your methods section, say that you used the SPIRITreporting guidelines, and cite them as:

Chan A-W, Tetzlaff JM, Altman DG, Laupacis A, Gøtzsche PC, Krleža-Jerić K, Hróbjartsson A, Mann H, Dickersin K, Berlin J, Doré C, Parulekar W, Summerskill W, Groves T, Schulz K, Sox H, Rockhold FW, Rennie D, Moher D. SPIRIT 2013 Statement: Defining standard protocol items for clinical trials. Ann Intern Med. 2013;158(3):200-207

		Reporting Item	Page Number
**Administrative information**			
Title	#1	Descriptive title identifying the study design, population, interventions, and, if applicable, trial acronym	1
Trial registration	#2a	Trial identifier and registry name. If not yet registered, name of intended registry	3
Trial registration: data set	#2b	All items from the World Health Organization Trial Registration Data Set	3
Protocol version	#3	Date and version identifier	15
Funding	#4	Sources and types of financial, material, and other support	16
Roles and responsibilities: contributorship	#5a	Names, affiliations, and roles of protocol contributors	1

For peer review only

Roles and responsibilities: sponsor contact information	#5b	Name and contact information for the trial sponsor	Appendix 1
Roles and responsibilities: sponsor and funder	#5c	Role of study sponsor and funders, if any, in study design; collection, management, analysis, and interpretation of data; writing of the report; and the decision to submit the report for publication, including whether they will have ultimate authority over any of these activities	Appendix 1
Roles and responsibilities: committees	#5d	Composition, roles, and responsibilities of the coordinating centre, steering committee, endpoint adjudication committee, data management team, and other individuals or groups overseeing the trial, if applicable (see Item 21a for data monitoring committee)	Appendix 1
**Introduction**			
Background and rationale	#6a	Description of research question and justification for undertaking the trial, including summary of relevant studies (published and unpublished) examining benefits and harms for each intervention	4
Background and rationale: choice of comparators	#6b	Explanation for choice of comparators	5
Objectives	#7	Specific objectives or hypotheses	6
Trial design	#8	Description of trial design including type of trial (eg, parallel group, crossover, factorial, single group), allocation ratio, and framework (eg,	6

For peer review only

		superiority, equivalence, non-inferiority, exploratory)	
**Methods: Participants, interventions, and outcomes**			
Study setting	#9	Description of study settings (eg, community clinic, academic hospital) and list of countries where data will be collected. Reference to where list of study sites can be obtained	7
Eligibility criteria	#10	Inclusion and exclusion criteria for participants. If applicable, eligibility criteria for study centres and individuals who will perform the interventions (eg, surgeons, psychotherapists)	7
Interventions: description	#11a	Interventions for each group with sufficient detail to allow replication, including how and when they will be administered	7
Interventions: modifications	#11b	Criteria for discontinuing or modifying allocated interventions for a given trial participant (eg, drug dose change in response to harms, participant request, or improving / worsening disease)	N/A. The experimental intervention (diagnostic aid) is an electronic tool that cannot be modified. If a practice wishes to withdraw from its use, it can do at any point of the trial. Parent/carers can opt out of its use and any data collection at the 6 week check.
Interventions: adherance	#11c	Strategies to improve adherence to intervention protocols, and any procedures for monitoring adherence (eg, drug tablet return; laboratory tests)	7

For peer review only

Interventions: concomitant care	#11d	Relevant concomitant care and interventions that are permitted or prohibited during the trial	N/A. The experimental intervention (diagnostic aid) is a low risk electronic checklist and does not affect any concomitant care
Outcomes	#12	Primary, secondary, and other outcomes, including the specific measurement variable (eg, systolic blood pressure), analysis metric (eg, change from baseline, final value, time to event), method of aggregation (eg, median, proportion), and time point for each outcome. Explanation of the clinical relevance of chosen efficacy and harm outcomes is strongly recommended	9
Participant timeline	#13	Time schedule of enrolment, interventions (including any run-ins and washouts), assessments, and visits for participants. A schematic diagram is highly recommended (see Figure)	Table 1
Sample size	#14	Estimated number of participants needed to achieve study objectives and how it was determined, including clinical and statistical assumptions supporting any sample size calculations	8
Recruitment	#15	Strategies for achieving adequate participant enrolment to reach target sample size	7
**Methods: Assignment of interventions (for controlled trials)**			

For peer review only

Allocation: sequence generation	#16a	Method of generating the allocation sequence (eg, computer-generated random numbers), and list of any factors for stratification. To reduce predictability of a random sequence, details of any planned restriction (eg, blocking) should be provided in a separate document that is unavailable to those who enrol participants or assign interventions	7
Allocation concealment mechanism	#16b	Mechanism of implementing the allocation sequence (eg, central telephone; sequentially numbered, opaque, sealed envelopes), describing any steps to conceal the sequence until interventions are assigned	7
Allocation: implementation	#16c	Who will generate the allocation sequence, who will enrol participants, and who will assign participants to interventions	7
Blinding (masking)	#17a	Who will be blinded after assignment to interventions (eg, trial participants, care providers, outcome assessors, data analysts), and how	15
Blinding (masking): emergency unblinding	#17b	If blinded, circumstances under which unblinding is permissible, and procedure for revealing a participant's allocated intervention during the trial	N/A. Trial participants will not be blinded.
**Methods: Data collection, management, and analysis**			
Data collection plan	#18a	Plans for assessment and collection of outcome, baseline, and other trial	9

For peer review only

		data, including any related processes to promote data quality (eg, duplicate measurements, training of assessors) and a description of study instruments (eg, questionnaires, laboratory tests) along with their reliability and validity, if known. Reference to where data collection forms can be found, if not in the protocol	
Data collection plan: retention	#18b	Plans to promote participant retention and complete follow-up, including list of any outcome data to be collected for participants who discontinue or deviate from intervention protocols	7
Data management	#19	Plans for data entry, coding, security, and storage, including any related processes to promote data quality (eg, double data entry; range checks for data values). Reference to where details of data management procedures can be found, if not in the protocol	14
Statistics: outcomes	#20a	Statistical methods for analysing primary and secondary outcomes. Reference to where other details of the statistical analysis plan can be found, if not in the protocol	11
Statistics: additional analyses	#20b	Methods for any additional analyses (eg, subgroup and adjusted analyses)	11
Statistics: analysis population and missing data	#20c	Definition of analysis population relating to protocol non-adherence (eg, as randomised analysis), and any statistical methods to handle missing data (eg, multiple imputation)	12

For peer review only

**Methods: Monitoring**			
Data monitoring: formal committee	#21a	Composition of data monitoring committee (DMC); summary of its role and reporting structure; statement of whether it is independent from the sponsor and competing interests; and reference to where further details about its charter can be found, if not in the protocol. Alternatively, an explanation of why a DMC is not needed	Appendix 1
Data monitoring: interim analysis	#21b	Description of any interim analyses and stopping guidelines, including who will have access to these interim results and make the final decision to terminate the trial	N/A. No interim analysis has been planned for this trial.
Harms	#22	Plans for collecting, assessing, reporting, and managing solicited and spontaneously reported adverse events and other unintended effects of trial interventions or trial conduct	16
Auditing	#23	Frequency and procedures for auditing trial conduct, if any, and whether the process will be independent from investigators and the sponsor	N/A. We have no plans for auditing this trial at the moment. the sponsor may conduct an audit at a random time. The clinical trial unit will conduct monthly central monitoring visits.
**Ethics and dissemination**			
Research ethics approval	#24	Plans for seeking research ethics committee / institutional review board (REC / IRB) approval	15

For peer review only

Protocol amendments	#25	Plans for communicating important protocol modifications (eg, changes to eligibility criteria, outcomes, analyses) to relevant parties (eg, investigators, REC / IRBs, trial participants, trial registries, journals, regulators)	16 and Appendix 1
Consent or assent	#26a	Who will obtain informed consent or assent from potential trial participants or authorised surrogates, and how (see Item 32)	7
Consent or assent: ancillary studies	#26b	Additional consent provisions for collection and use of participant data and biological specimens in ancillary studies, if applicable	N/A. No collection of samples in this trial.
Confidentiality	#27	How personal information about potential and enrolled participants will be collected, shared, and maintained in order to protect confidentiality before, during, and after the trial	9
Declaration of interests	#28	Financial and other competing interests for principal investigators for the overall trial and each study site	16
Data access	#29	Statement of who will have access to the final trial dataset, and disclosure of contractual agreements that limit such access for investigators	Appendix 1
Ancillary and post trial care	#30	Provisions, if any, for ancillary and post-trial care, and for compensation to those who suffer harm from trial participation	N/A. No post trial care is expected. The respective information sheets contain details of Patient Advice Liasion (PALs) services that participants can contact for complaints and compensation.

For peer review only

Dissemination policy: trial results	#31a	Plans for investigators and sponsor to communicate trial results to participants, healthcare professionals, the public, and other relevant groups (eg, via publication, reporting in results databases, or other data sharing arrangements), including any publication restrictions	15
Dissemination policy: authorship	#31b	Authorship eligibility guidelines and any intended use of professional writers	N/A. These have not yet been drawn up.
Dissemination policy: reproducible research	#31c	Plans, if any, for granting public access to the full protocol, participant-level dataset, and statistical code	15
**Appendices**			
Informed consent materials	#32	Model consent form and other related documentation given to participants and authorised surrogates	N/A. This trial is multifaceted and thus has many information sheets and consent forms for different aspects of the trial.
Biological specimens	#33	Plans for collection, laboratory evaluation, and storage of biological specimens for genetic or molecular analysis in the current trial and for future use in ancillary studies, if applicable	N/A. No collection of samples required for this trial.

None The SPIRIT checklist is distributed under the terms of the Creative Commons Attribution License CC-BY-ND 3.0. This checklist can be completed online using https://www.goodreports.org/, a tool made by the EQUATOR Network in collaboration with Penelope.ai

For peer review only

BMJ Open

Study protocol for evaluation of aid to diagnosis for developmental dysplasia of the hip in general practice: controlled trial randomised by practice

Journal:	*BMJ Open*
Manuscript ID	bmjopen-2020-041837.R2
Article Type:	Protocol
Date Submitted by the Author:	11-Nov-2020
Complete List of Authors:	Roposch, Andreas; UCL, Institute of Child Health; Great Ormond Street Hospital for Children, Department of Orthopaedic Surgery<br>warsame, kaltuun; University College London Institute of Child Health,<br>Chater, Angel; University of Bedfordshire, Department of Sport Science and Physical<br>Green, Judith; Kings College London<br>Hunter, Rachael; University College London, Research Dept of Primary Care and Population Health<br>Wood, J; University College London, PRIMENT Clinical Trials Unit<br>Freemantle, Nick; University College London, Comprehensive Clinical Trials Unit<br>Nazareth, Irwin; UCL, Primary Care and Population Health
**Primary Subject Heading**:	General practice / Family practice
Secondary Subject Heading:	Qualitative research, Public health, Paediatrics
Keywords:	PRIMARY CARE, Paediatric orthopaedics < ORTHOPAEDIC & TRAUMA SURGERY, PAEDIATRICS, PUBLIC HEALTH

SCHOLARONE™
Manuscripts

I, the Submitting Author has the right to grant and does grant on behalf of all authors of the Work (as defined in the below author licence), an exclusive licence and/or a non-exclusive licence for contributions from authors who are: i) UK Crown employees; ii) where BMJ has agreed a CC-BY licence shall apply, and/or iii) in accordance with the terms applicable for US Federal Government officers or employees acting as part of their official duties; on a worldwide, perpetual, irrevocable, royalty-free basis to BMJ Publishing Group Ltd ("BMJ") its licensees and where the relevant Journal is co-owned by BMJ to the co-owners of the Journal, to publish the Work in this journal and any other BMJ products and to exploit all rights, as set out in our licence.

The Submitting Author accepts and understands that any supply made under these terms is made by BMJ to the Submitting Author unless you are acting as an employee on behalf of your employer or a postgraduate student of an affiliated institution which is paying any applicable article publishing charge ("APC") for Open Access articles. Where the Submitting Author wishes to make the Work available on an Open Access basis (and intends to pay the relevant APC), the terms of reuse of such Open Access shall be governed by a Creative Commons licence – details of these licences and which Creative Commons licence will apply to this Work are set out in our licence referred to above.

Other than as permitted in any relevant BMJ Author's Self Archiving Policies, I confirm this Work has not been accepted for publication elsewhere, is not being considered for publication elsewhere and does not duplicate material already published. I confirm all authors consent to publication of this Work and authorise the granting of this licence.

For peer review only

Study Protocol for evaluation of aid to diagnosis for developmental dysplasia of the hip in general practice: controlled trial randomised by practice

Andreas Roposch, Kaltuun Warsame, Angel Chater, Judith Green, Rachael Hunter, John Wood, Nick Freemantle, Irwin Nazareth

Author Affiliations

Andreas Roposch MD MSc FRCS; Kaltuun Warsame BSc

UCL Great Ormond Street Institute of Child Health

30 Guilford Street, London WC1N 3EH

Angel Chater PhD

Faculty of Education and Sport, University of Bedfordshire

Polhill Avenue, Bedford MK41 9EA

Judith Green PhD

Department of Population Health Sciences, Kings College London

Great Maze Pond, London SE1 1UL

Nick Freemantle PhD

Institute of Clinical Trials and Methodology, University College London

90 High Holborn, London WC1V 6LJ

Irwin Nazareth PhD FRCGP; Rachael Hunter MSc; John Wood MSc

Research Department for Primary Care and Population Health, University College London

Rowland Hill Street, London NW3 2PF

Corresponding author: Professor Andreas Roposch, UCL Great Ormond Street Institute of Child Health, 30 Guilford Street, London WC1N 3EH - a.roposch@ucl.ac.uk

Keywords: Developmental dysplasia of the hip, primary care, diagnostic aid

For peer review only

ABSTRACT

Introduction: In the UK, a compulsory '6-week hip check' is performed in primary care for the detection of developmental dysplasia of the hip (DDH). However, missed diagnoses and infants incorrectly labelled with DDH remain a problem, potentially leading to adverse consequences for infants, their families and the NHS. National policy states that infants should be referred to hospital if the 6-week check suggests DDH, though there is no available tool to aid examination or offer guidelines for referral. We developed standardised diagnostic criteria for DDH, based on international Delphi consensus, and a 9-item checklist that has the potential to enable non-experts to diagnose DDH in a manner approaching that of experts.

Methods and Analysis*:* We will conduct a controlled trial randomised by practice that will compare a diagnostic aid against standard care for the hip check. The primary objective is to determine whether an aid to the diagnosis of DDH reduces the number of clinically insignificant referrals from primary care to hospital, and the number of late diagnosed DDH. The trial will include a qualitative process evaluation, an assessment of professional behaviour change and a full health economic evaluation. We will recruit 152 GP practices in England. These will be randomised to conduct the hip checks with use of the study diagnostic aid and/or as per usual practice. The total number of infants seen during a 15-month recruitment period will be 110 per practice. Two years after the 6-week hip check we will measure the number of referred infants that are (1) clinically insignificant for DDH and (2) those that constitute appropriate referrals.

Ethics and Dissemination: This study has approval from the Health Research Authority (16/1/2020) and the Confidentiality Advisory Group (18/2/2020). Results will be published in peer-reviewed academic journals, disseminated to patient organisations and the media.

Registration details: clinicaltrials.gov (NCT04101903; Pre-results).

For peer review only - http://bmjopen.bmj.com/site/about/guidelines.xhtml

STRENGTHS AND LIMITATIONS OF THIS STUDY

- To our knowledge this is the first trial to evaluate a diagnostic aid for the 6-week check with reference to evaluating both missed and unnecessary referrals to hospital.
- Implementable on existing clinical software used by GPs, the proposed aid will be easy to use.
- A comprehensive process evaluation, using qualitative methods and behaviour change frameworks, will be done alongside to the trial; plus a full health economic evaluation.
- The reliance on existing practice staff to report monthly updates is essential but could pose a risk to timely and complete data collection.
- The collection of identifiable data through site visits across 152 practices will be challenging.

INTRODUCTION

Developmental dysplasia of the hip (DDH) is characterized by varying displacement of the proximal femur from the acetabulum with associated acetabular dysplasia. Dislocation occurs in 1-2/1000 infants per year but milder forms occur in 40-60/1000.[1] Early recognition of disease is associated with better outcomes. It is national policy[2] to examine all infants for the presence of DDH at birth and at age 6-8 weeks in primary care (6-week hip check). If diagnosed within the first 6-8 weeks, splinting of the hips is successful in 85% of cases.[3] Later diagnoses usually will require invasive treatment, with many years of continued monitoring.[4] Late diagnosed DDH is a common cause of medical negligence claims, with increased suffering for affected patients.

Timely diagnoses of DDH remain a challenge despite the compulsory 6-week check.[5] In one study, the median age at diagnosis was 14 months, with only 40% of infants diagnosed during routine examinations and 60% presenting owing to parental concerns.[6] In another study, 30% of infants were not diagnosed by 12 weeks.[7] Because there is no further compulsory check after that at 6-weeks, it is vital that the 6-week check is effective. Also infants incorrectly identified with "DDH" in primary care and referred to hospital remain a challenge for both families and NHS. This group does not

For peer review only

require treatment and reassurance provided in primary care would avoid unnecessary anxiety and inefficient use of hospital resources. Of 1,918 infants referred to hospital from primary care for DDH, only 64 (3%) had DDH[7] but 1,270 (66%) were identified as 'DDH' based on inappropriate criteria,[8] e.g. "crease asymmetry" (n=234) or "click" (n=648). If GPs were able to discriminate better between benign abnormalities requiring reassurance (perhaps with a follow-up in primary care) and findings requiring referrals, outcomes of infants with *and without* DDH would improve.

In prior research we suggested that too many diagnostic criteria[8] and variability in the use of diagnostic criteria among clinicians[9] complicate the task of diagnosing DDH. We developed standardised diagnostic criteria to reduce the variability in assessment and management decisions in infants examined for DDH.[10] The weighted criteria demonstrated validity.[10] In a study of 44 patients referred from GPs to hospital, the weighted criteria demonstrated a positive predictive value of 89% (95% confidence interval 70, 97) and negative predictive value of 76% (95% confidence interval, 50-96).[11] We refined these binary criteria, in form of a checklist with 9 items, for use by GPs during the 6-week hip check and developed a training video, featuring a GP. This checklist and video were refined in a feasibility study which used qualitative methods to explore the acceptability of the format, style and delivery of the intervention (report available on request). The video explains the meaning of the diagnostic criteria; it also demonstrates how precisely to elicit the diagnostic criteria. For example, the video explains the difference between the Barlow and Ortolani manoeuvres, how to test for a leg length inequality, or how to identify limitations in hip abduction.

Rationale

Current referral patterns suggest there could be considerable health gains from improved diagnostic and referral decisions at the 6-week check.[5] GPs, in a preparatory focus group, identified the need for a 'comprehensive, structured guide' for the 6-week check. Diagnostic aids enable physicians to overcome barriers in diagnostic reasoning[12] by shifting intuitive to analytical aspects of diagnostic reasoning. Decisions made under these circumstances approach normative reasoning and rationality

For peer review only

more closely, and are more reliable and safer.[13] Building on our earlier research, we propose to facilitate GP's diagnostic reasoning at the 6-week check by structuring current practice with use of a previously developed diagnostic aid. A diagnostic aid of this kind provides a structured approach to the assessment of infants and offers guidance about referrals.

Objectives

- To determine whether an aid to the diagnosis of DDH reduces the number of clinically insignificant referrals from primary care to hospital, and the number of late diagnosed DDH.
- To determine the cost-effectiveness of this intervention.
- To conduct an integrated qualitative and quantitative process evaluation in order to understand all participants' experience in the trial and with the intervention; study how the intervention is implemented; investigate contextual factors that affect the intervention.

METHODS AND ANALYSIS

The SPIRIT reporting guidelines were used in the preparation of this clinical trial protocol.[14]

Design

This is a cluster randomised controlled trial of GP practices in England, with randomisation and intervention at practice level but the primary endpoint measured at patient level (Figure 1). An internal pilot will be done in the first 4 months to ascertain accrual rates – we will progress to the main trial if we succeed in recruiting ≥12 practices per month (expected recruitment is ≥21 practices/month for the main trial). Incorporated in the trial are (i) a process evaluation investigating determinants of GPs' referral behaviour and the implementation of the intervention in practice; and (ii) a health economic evaluation in a child's lifetime.

Eligibility and recruitment

For peer review only - http://bmjopen.bmj.com/site/about/guidelines.xhtml

GP practices registered in England that carry out 6-week hip checks (at age 42-76 days) and agree (i) to being randomised and (ii) to hospitals releasing data concerning infants they examine in the trial. Ineligible are practices planning to close with 12 months of trial start.

We anticipate that ≥110 infants per year will undergo the 6-week hip check in each of the recruited practices. Each practice will recruit infants for a period of 15 months and infants will be followed for 2 years. This trial is planned therefore to run for 44 months across 152 GP practices.

Retention

The proposed trial is not onerous on the practice staff and practices will not be challenged by excessive study-related manoeuvres. Because the primary outcome will be collected using practice and hospital databases only, it is likely that we will attain high levels of follow up. We will send periodic newsletters to GPs and foster regular contacts.

Randomisation

Practices will submit an eligibility form to the clinical trials unit, which will register and randomly allocate (concealed) practices within 48 hours. An independent statistician will coordinate randomisation with an allocation ratio of 1:1 and stratified by practice size (based on observed sizes obtained from expressions of interest – we expect 2 or 3 strata).

Interventions

Control intervention: GPs will assess infant hips following best practice principles; they will be provide a leaflet about the national best practice recommendations.[2]

Experimental intervention: This is a complex intervention comprising a video developed specifically for this trial and a diagnostic aid in form of a 9-item binary checklist. At the time when informed consent is taken, GPs will watch the video. They will then examine all infants in the 6-week check according to best practice principles, but with the addition of the diagnostic aid (implemented electronically to each practice's computer system).

For peer review only

According to national guidelines, the 6-week check shall capture infants at age 6-10 weeks and infants in whom a diagnosis of DDH is considered should see a specialist within 2 weeks.[2] Participating GPs of both intervention arms will be asked to adhere to this policy.

Definitions for the purpose of this trial

'Appropriate referral' denotes a referred hip deemed 'clinically significant', i.e. is treated or monitored (at least one follow-up) by a specialist surgeon. Any ambiguous cases will be reviewed by our Expert Advisory Panel who will assign an ultimate diagnosis in consensus. 'Clinically insignificant' are referred hips resulting in reassurance and discharge from surgeons' clinics (i.e. no treatment or monitoring). 'Late diagnoses' are cases of DDH diagnosed by a specialist surgeon at age 3-24 months.[15;16]

Sample size calculation

We based its calculation on Poisson regression and audit data from the Nottingham area (partially presented in Price et al[7]) and University College Hospital (audit of 3 years' practice, unpublished data): a conservative estimate of the number of infants referred as a consequence of the 6-week check within the recruitment period of one year is, on average, 3 per practice. Based on the same data, we estimate that about 1 in 3 (or, on average, 1 per practice) of such referrals is correctly made ('appropriate' referrals, defined above as 'clinically significant', are a subset of these 'correct' referrals). Thus, on average, we expect 2 'incorrect' referrals per practice. These are the ones we seek to reduce, and the intervention should serve to cut them by 50%. Thus we aim to detect a reduction, on average, from 3 to 2 referrals per practice in the intervention arm. To account for correlation within practices, which can also be thought of as overdispersion relative to the underlying Poisson variation, we assumed a between-practice component of variance of 20% of the average Poisson 'counting' variance per practice. For 90% statistical power and testing 2-sided at p=0.05, we need 76 practices per group (15% of this total comprises a safety margin to allow for potential challenges). Average referral rates from the Nottingham (2.8%) and University College Hospital data

For peer review only - http://bmjopen.bmj.com/site/about/guidelines.xhtml

(2.7%) are very consistent, and suggest a target of about 110 children per practice per year to undergo the 6-week check.

Primary trial endpoint

Number of referred infants that are considered 'clinically insignificant' – this is a measure of the clinical importance of referrals in the 2-week hip pathway. We chose this outcome because it is extremely relevant clinically and one for which it is possible to power the trial.[2] An intervention could be successful at achieving this outcome whilst missing infants who do have DDH; we thus specified a *principal secondary endpoint*. False negatives are also important but, because of their rarity, would not be an efficient endpoint on which to power the trial (and would thus be outside ICH E9,[17] which recommends that the primary outcome should be both clinically and statistically convincing). A research assistant will retrieve these data from respective hospital electronic systems by conducting site visits to all secondary care facilities to which GPs refer infants, starting 6 weeks after the last patient enters the trial.

Secondary trial endpoints (Table 1)

Infant level

• Number of appropriate referrals per practice (principal secondary endpoint): for all infants referred as a consequence of the 6-week check, a researcher will collect, using unique NHS patient numbers, and categorise the appropriateness of referrals by a practice with respective hospital databases using a standardised taxonomy, blind to practice random allocation. S/he will conduct site visits to all such hospitals starting 6 weeks after the last patient entered the trial.

• Number of late diagnoses: We will employ deterministic methods of data linkage. Using unique NHS numbers of any recruited infant, we will obtain from Health & Social Care Information Centre the corresponding Health Episodes Statistic (HES) identifiers. HES is a data warehouse that includes all hospital admissions and outpatients' visits occurring in all hospitals in England. In HES we will establish whether any infant in the trial was admitted to a UK hospital as a result of DDH using

For peer review only - http://bmjopen.bmj.com/site/about/guidelines.xhtml

relevant ICD-10 codes and OPCS-4 codes.[18] We will extract, for the whole trial period, the full hospital history for all infants in the trial (in and out patient episodes), collate all episodes into a combined exploratory analysis view, collate interactions, and allocate outcomes to practice groups to facilitate outcome analyses.[19] These data will be compared with the data collected by the researcher (see above); the use of these 2 strategies will enhance the robustness of data.

• Consequences of late diagnoses: Using data collected above we will record nature and frequency of such consequences (nature of treatment, length of hospital stay, frequency of secondary care contacts).

• Health-related quality of life (parental proxy report): Child-Health-Utility-9D.[20]

Process level

• Volume of referrals: Total number of patients referred to secondary care during the trial period. This variable will be collected prospectively at practice level by the participating GP and forwarded monthly to the clinical trial unit.

• Timeliness of referrals: In infants referred to secondary care, we will measure the days from referral issued to hospital appointment (collected in the same way as primary endpoint). This process measure will inform about target wait times[2] achieved.

Clinician level

• Confidence and attitudes of GP and secondary care clinicians towards the diagnostic aid: will be assessed 12 weeks after trial set-up and at trial completion using a modified measure based on the Theory of Planned Behaviour.[21]

• Implementation issues and acceptability of diagnostic aid among GPs and secondary care providers: we will conduct qualitative research at the trial end to elicit this information.

• Use of diagnostic aid and acceptability of intervention: a self-administered questionnaire to evaluate the use of the aid will be posted to all the GPs in the intervention group; also direct observations and interviews will ascertain this outcome.

For peer review only - http://bmjopen.bmj.com/site/about/guidelines.xhtml

For peer review only

Parent/carer level (collected from one in ten parents/carers)

• General worry: State-Trait Anxiety Inventory 6-items short form.[22]

• DDH-related worry: Infant Hip Worries Inventory.[23]

• Satisfaction with trial: dimensions of care items from EUROPEP.[24]

For peer review only

For peer review only - http://bmjopen.bmj.com/site/about/guidelines.xhtml

Table 1. Assessments at different time points

	**Baseline**	**Within 2 weeks of consent**	**At 6-week hip check**	**3-month follow-up**	**15-month follow-up**	**2-year follow-up**
Clinician-level						
Theoretical Domains Framework questionnaire	X		X	X	X	X
GP characteristics questionnaire	X					
Fidelity questionnaire			X	X	X	X
Non-participant observations			X			
Semi-structured interviews			X		X	
Parent/carer-level						
State Trait Anxiety Inventory 6 item short form			X			
Infant Hip Worries Inventory			X			
EUROPEP			X			
Non-participant observations			X			
Semi-structured interviews		X				
Out-of-pocket-costs questionnaire			X			
EuroQol 5 Dimension Scale (EQ-5D)			X			X
Child Health Utility 9D (CHU 9D)						X
Willingness-to-pay questionnaire					X	

For peer review only

Statistical analysis

Primary endpoint: We will compare the randomised practices using Poisson mixed models, accounting for extra *Poissonian* variability by including random intercept terms for practices. The response variable will be the number of clinically insignificant referrals from each practice with an offset in the linear model of the log(e) total number of children checked in each practice, to account for differences in practice size and constitution. The random effect at the practice level will account for overdispersion.

Principal secondary endpoint: This will be analysed as above; however, counts here are expected to be lower. Should they be insufficient to support the full model, the model will be simplified by (e.g. use of additive variance component term rather than a generalised random intercept term to address overdispersion). We will describe the planned statistical analysis and its adaptation where there are difficulties in achieving convergence in a pre-specified statistical analysis plan. No interim analyses are planned.

Late diagnoses: As we anticipate small numbers of events in each randomised comparison we may not be in a position to account for practices using random intercept terms. Where this is the case we will report the total numbers over a minimum 2-year observation and compare the overall group scores using Fishers exact test.

Quantitative outcome measures: We will summarize scores by instrument, accounting for practices and report difference in group means for each treatment arm. We expect mean scores to be lower in the intervention arm for State-Trait Anxiety Inventory and Infant Hip Worries Inventory, but higher or equal for EUROPEP.

Missing data: For the primary endpoint and principal secondary endpoint the data collection methods should identify qualifying episodes. Because of the nature of these data the conventional concept of missingness does not directly apply (eg we will not have individually randomised subjects who cannot be followed up). However if a practice withdraws from the trial we will explore the

For peer review only

consequences of this action by assuming a poor outcome among that practice if in the intervention group and a good outcome if in the control condition, to identify the potential consequences of their withdrawal. Complete case analyses will be conducted for secondary outcomes. If there is a mismatch between practices in the two treatment conditions we will consider undertaking joint models examining simultaneously the binomial of missingness and the outcome measure of interest.

Health economics

Cost analysis

We will analyse the cost associated with the intervention compared with usual practice for the entire trial period, and examine costs from the perspective of the NHS and of families. The cost components included in main analysis are: cost of 6-week in both intervention arms; any subsequent referrals, diagnostic tests and treatment. We will collect costs about GP time, which we will multiply by unit costs from routine sources.[25]

Within-trial cost-effectiveness analysis

With the costs described above we will produce a dataset of patient-level within-trial costs and outcomes. We will calculate the incremental cost per clinically insignificant referral avoided and the incremental cost per late diagnosis avoided. Using bootstrapping of the mean cost and outcomes differences, we will estimate confidence intervals around the incremental cost-effectiveness ratios.[26] With the bootstrap replications we will construct a cost-effectiveness acceptability curve to show the probability that the aid is cost-effective for different values of NHS willingness to pay for outcomes. We will perform deterministic sensitivity analyses.

Long-run cost utility analysis

We will use several measures to evaluate the lifetime cost-effectiveness of the intervention. We will ask 20 carers of infants aged 2-4 years to complete (1) on behalf of their children the Child-Health-utility-9D[20] and (2) for themselves the EQ-5D-5L,[27] both measure health-related quality of life.

Cost-benefit analysis

For peer review only - http://bmjopen.bmj.com/site/about/guidelines.xhtml

With data from the trial about the impact of the intervention on appropriate referrals, we will calculate the monetary value that parents place on the intervention using willingness-to-pay methodology.[28] This will provide an estimate of the monetary value of the additional benefits (positive/negative) of the intervention. We will calculate the net benefit of the intervention by subtracting the incremental cost of the aid, as calculated above from the trial data, from the monetary value of the additional benefit. Following recruitment of the last infant in the trial, we will recruit 200 carers of infants undergoing the 6-week check from trial-participating practices. These will be 100 carers whose infants will be referred to secondary care as a consequence of the 6-week check and 100 who will not. They will complete a self-report questionnaire that utilised several techniques[28] to elicit willingness-to-pay values. We will calculate willingness-to-pay values for the whole sample and test for variations based on socio-demographic groups and referral to secondary care.

Integrated qualitative and quantitative process evaluation

This workstream will explore the implementation, adherence to protocol, receipt and setting of the intervention. We will examine the views of all groups of participants on the intervention; study how the intervention is implemented; investigate contextual factors that affect the intervention; and study how effects vary in subgroups of GPs. These data will help in understanding how, for whom and why the trial had effects and the extent to which outcomes result from issues of trial fidelity and implementation. We will collect process data from all 152 sites including clinician and carer outcomes. We will conduct alongside the trial non-participant observation and semi-structured interviews. We will include a purposive sample of 10 practices for the qualitative study, interviewing 4-5 participants in each (e.g. GP, carers, hospital consultant), resulting in 40-50 interviews. This sample will include a small number of practices in the control arm (for comparative purposes), and a range of intervention practices to include different locations, practice sizes and types. We will analyse process data before outcome data to avoid bias in interpretation.[29] Interviews will be audio-

For peer review only - http://bmjopen.bmj.com/site/about/guidelines.xhtml

recorded and transcribed, data from observations will be recorded contemporaneously using a template. Data from process outcomes, interviews (transcripts) and observations will be analysed from the perspective of both behaviour change theory[30] and normalization process theory.[31]

Strategies to mitigate potential bias

Since this effectiveness trial will test whether the intervention can work under usual circumstances, we will rely on paediatric orthopaedic surgeons in determining the ultimate diagnosis of DDH. Variations in the surgeons' diagnostic accuracy are inevitable hence the need for a randomised study. We will perform analyses by surgeon (or hospital) to quantify this variation. Blinding of GPs, practice staff, carers is impossible; however, most such outcomes will be assessed with validated questionnaires. Primary and principal secondary endpoints will be collected by an independent researcher blinded to treatment allocation. In case an infant is referred to hip ultrasound without an orthopaedic consultation, a trial-appointed advisory panel shall review the scan blinded and according to standard methods[32] to avoid reporting bias. There is a risk for verification bias – while our trial includes a 2-year follow up to capture late presenting DDH, we cannot rule out that some infants with DDH will remain undiagnosed within this period, thus underestimating the number of late diagnosed DDH. However, the 2-year mark has previously been found to be a robust outcome.[33]

Patient and public involvement

We developed this protocol with carers of children with DDH and the founding director of 'Steps', a charity supporting patients with lower limb disorders. We discussed the need for the trial and trial procedures and conduct with staff members of GP practices. Our established patient and public involvement group has reviewed and commented on this protocol and will periodically review, support and advise on the conduct of the trial.

Trial and data management

The trial will be run through PRIMENT Clinical Trials Unit and conducted in accordance with established quality management systems and standardised operating procedures (Appendix 1). All

For peer review only - http://bmjopen.bmj.com/site/about/guidelines.xhtml

data will be handled in accordance with the UK Data Protection Act 2018. All analyses will be conducted blinded to allocation groups.

Ethics and dissemination

Leicester Central Research Ethics Committee (19/EM/0317, 16/1/2020) and Health Research Authority Confidentiality Advisory Group (19/CAG/0198, 18/2/2020) approved protocol version 2. 'Section 251' approval was obtained, which omits the need for written informed consent from parents/carers; consent will be obtained at cluster level from the lead GPs. This trial is registered at clinicaltrials.gov (NCT04101903). We will publish results in peer-reviewed journals and disseminate results to patient organisations and the media.

Discussion

This trial is part of a programme of research to improve the diagnosis of DDH: consensus-based diagnostic criteria were established in prior research, tailored for use in primary care, supplemented by a video designed for GPs. There has only been one randomised trial on the topic of DDH in the UK[33] but it explored the use of ultrasound screening – our trial focusses on the compulsory '6-week check'. Because the intervention tested in this trial is based on consensus of clinical experts, there is a risk that the opinions of experts change as clinical knowledge evolves. However, the criteria of the diagnostic aid have been in use for decades and will likely not loose relevance in the foreseeable future. The collection of outcome data from various hospitals connected to GP practices will be challenging; use of national health services databases should mitigate this challenge. While our trial includes a 2-year followup period to capture late presenting DDH, we cannot rule out that some infants with DDH will remain undiagnosed within this period. This trial has the potential to improve the compulsory 6-week hip check with use of a relatively simple intervention. It will also provide an understanding of the cost effectiveness of the intervention in a whole lifetime horizon of a 6-week old. If successful, the intervention can be rolled out to clinical services relatively easily and at low costs.

For peer review only - http://bmjopen.bmj.com/site/about/guidelines.xhtml

Author contributions

AR, IN and NF were involved in conception and trial design. JG expanded and developed the qualitative process evaluation of the protocol. AC designed the behaviour change psychology aspects of the trial, working closely with JG and AR. RH, AR, IN developed the health economic evaluation plan. JW, IN, AR, IN developed all statistical aspects of the trial. AR and KW drafted the manuscript for publication. IN, JG, AC, IN, JW, NF, RH, KW and AR critically revised the article for important intellectual content and approved the final version of the manuscript. AR is chief investigator of the trial and together with IN, NF, JW, JG, AC secured its funding. We acknowledge the valuable input from Professor Daniel Ray in relation to the collaborative work with NHS Digital for outcome data collection.

Funding statement

This paper presents independent research funded by the National Institute for Health Research (NIHR) under its Programme Grant for Applied Research funding stream (RP-PG-0616-20006). The views expressed are those of the authors and not necessarily those of the NIHR or the Department of Health and Social Care.

Competing interest statement

None

References

(1) Bialik V, Bialik GM, Blazer S, Sujov P, Wiener F, Berant M. Developmental dysplasia of the hip: a new approach to incidence. *Pediatrics* 1999; 103(1):93-99.

(2) Public Health England. Newborn and infant physical examination (NIPE) sceening handbook. www.gov.uk/government/publications/newborn-and-infant-physical-examination-programme-handbook/newborn-and-infant-physical-examination-screening-programme-handbook . 27-8-2019.

For peer review only - http://bmjopen.bmj.com/site/about/guidelines.xhtml

(3) Clarke NM, Reading IC, Corbin C, Taylor CC, Bochmann T. Twenty years experience of selective secondary ultrasound screening for congenital dislocation of the hip. *Arch Dis Child* 2012; 97(5):423-429.

(4) NJR Editorial Board. 10th Annual report: national joint registry for England, Wales and Northern Ireland. 2013.

(5) Davies R, Talbot C, Paton R. Evaluation of primary care 6- to 8-week hip check for diagnosis of developmental dysplasia of the hip: a 15-year observational cohort study. *Br J Gen Pract* 2020; 70(693):e230-e235.

(6) Sanghrajka AP, Murnaghan CF, Shekkeris A, Eastwood DM. Open reduction for developmental dysplasia of the hip: failures of screening or failures of treatment? *Ann R Coll Surg Engl* 2013; 95(2):113-117.

(7) Price KR, Dove R, Hunter JB. Current screening recommendations for developmental dysplasia of the hip may lead to an increase in open reduction. *Bone Joint J* 2013; 95-B(6):846-850.

(8) Roposch A, Liu LQ, Hefti F, Clarke NM, Wedge JH. Standardized diagnostic criteria for developmental dysplasia of the hip in early infancy. *Clin Orthop Relat Res* 2011; 469(12):3451-3461.

(9) Roposch A, Liu LQ, Protopapa E. Variations in the use of diagnostic criteria for developmental dysplasia of the hip. *Clin Orthop Relat Res* 2013; 471(6):1946-1954.

(10) Roposch A, Protopapa E, Cortina-Borja M. Weighted diagnostic criteria for developmental dysplasia of the hip. *J Pediatrics* 2014; 165:1236-1240.

(11) Roposch A, Protopapa E. Testing newly developed standardized diagnostic criteria for DDH in consecutive patients referred to a paediatric orthopaedic unit. *Pediatric Orthopedic Society of North America* 2013.

(12) El-Kareh R, Hasan O, Schiff GD. Use of health information technology to reduce diagnostic errors. *BMJ Qual Saf* 2013; 22 Suppl 2:ii40-ii51.

(13) Croskerry P. Clinical cognition and diagnostic error: applications of a dual process model of reasoning. *Adv Health Sci Educ Theory Pract* 2009; 14 Suppl 1:27-35.

(14) Chan AW, Tetzlaff JM, Altman DG, Laupacis A, Gotzsche PC, Krleza-Jeric K et al. SPIRIT 2013 statement: defining standard protocol items for clinical trials. *Ann Intern Med* 2013; 158(3):200-207.

(15) Woolacott NF, Puhan MA, Steurer J, Kleijnen J. Ultrasonography in screening for developmental dysplasia of the hip in newborns: systematic review. *BMJ* 2005; 330(7505):1413.

(16) Clinical practice guideline: early detection of developmental dysplasia of the hip. Committee on Quality Improvement, Subcommittee on Developmental Dysplasia of the Hip. American Academy of Pediatrics. *Pediatrics* 2000; 105(4 Pt 1):896-905.

For peer review only - http://bmjopen.bmj.com/site/about/guidelines.xhtml

(17) ICH Steering Committee. ICH harmonised tripartite guideline: Statistical principles for clinical trials. https://database.ich.org/sites/default/files/E9_Guideline . 5-2-1998.

(18) NHS Digital. National clinical coding standards OPCS-2 (2018). Norwich, England: TSO Shop; 2018.

(19) MacArthur C, Jolly K, Ingram L, Freemantle N, Dennis CL, Hamburger R et al. Antenatal peer support workers and initiation of breast feeding: cluster randomised controlled trial. *BMJ* 2009; 338:b131.

(20) Stevens K. Assessing the performance of a new generic measure of health-related quality of life for children and refining it for use in health state valuation. *Appl Health Econ Health Policy* 2011; 9(3):157-169.

(21) Powell AA, Bloomfield HE, Burgess DJ, Wilt TJ, Partin MR. A conceptual framework for understanding and reducing overuse by primary care providers. *Med Care Res Rev* 2013; 70(5):451-472.

(22) Marteau TM, Bekker H. The development of a six-item short-form of the state scale of the Spielberger State-Trait Anxiety Inventory (STAI). *Br J Clin Psychol* 1992; 31 (Pt 3):301-306.

(23) Gardner F, Dezateux C, Elbourne D, Gray A, King A, Quinn A et al. The hip trial: psychosocial consequences for mothers of using ultrasound to manage infants with developmental hip dysplasia. *Arch Dis Child Fetal Neonatal Ed* 2005; 90(1):F17-F24.

(24) Wensing M. EUROPEP 2006: Revised EUROPEP instrument and user manual. www.equip.ch/files/25/europep_2006rapport.pdf 6-6-2014.

(25) Department of Health. Department of Health. National Schedule of Reference Costs - Year 2011-12 - NHS Trusts and NHS Foundation Trusts: NHS own costs. 2015.

(26) Briggs AH, Wonderling DE, Mooney CZ. Pulling cost-effectiveness analysis up by its bootstraps: a non-parametric approach to confidence interval estimation. *Health Econ* 1997; 6(4):327-340.

(27) Herdman M, Gudex C, Lloyd A, Janssen M, Kind P, Parkin D et al. Development and preliminary testing of the new five-level version of EQ-5D (EQ-5D-5L). *Qual Life Res* 2011; 20(10):1727-1736.

(28) McIntosh E. Using discrete choice experiments within a cost-benefit analysis framework: some considerations. *Pharmacoeconomics* 2006; 24(9):855-868.

(29) Oakley A, Strange V, Bonell C, Allen E, Stephenson J. Process evaluation in randomised controlled trials of complex interventions. *BMJ* 2006; 332(7538):413-416.

(30) Michie S, Richardson M, Johnston M, Abraham C, Francis J, Hardeman W et al. The behavior change technique taxonomy (v1) of 93 hierarchically clustered techniques: building an international consensus for the reporting of behavior change interventions. *Ann Behav Med* 2013; 46(1):81-95.

For peer review only - http://bmjopen.bmj.com/site/about/guidelines.xhtml

(31) Murray E, Treweek S, Pope C, MacFarlane A, Ballini L, Dowrick C et al. Normalisation process theory: a framework for developing, evaluating and implementing complex interventions. *BMC Med* 2010; 8:63.

(32) Roposch A, Moreau NM, Uleryk E, Doria AS. Developmental dysplasia of the hip: quality of reporting of diagnostic accuracy for US. *Radiology* 2006; 241(3):854-860.

(33) Elbourne D, Dezateux C, Arthur R, Clarke NMP, Gray A, King A et al. Ultrasonography in the diagnosis and management of developmental hip dysplasia (UK Hip Trial): clinical and economic results of a multicentre randomised controlled trial. *Lancet* 2002; 360(9350):2009-2017.

For peer review only

Figure legends

Figure 1. Flowchart demonstrating pathways and principal endpoints and their collection.

For peer review only - http://bmjopen.bmj.com/site/about/guidelines.xhtml

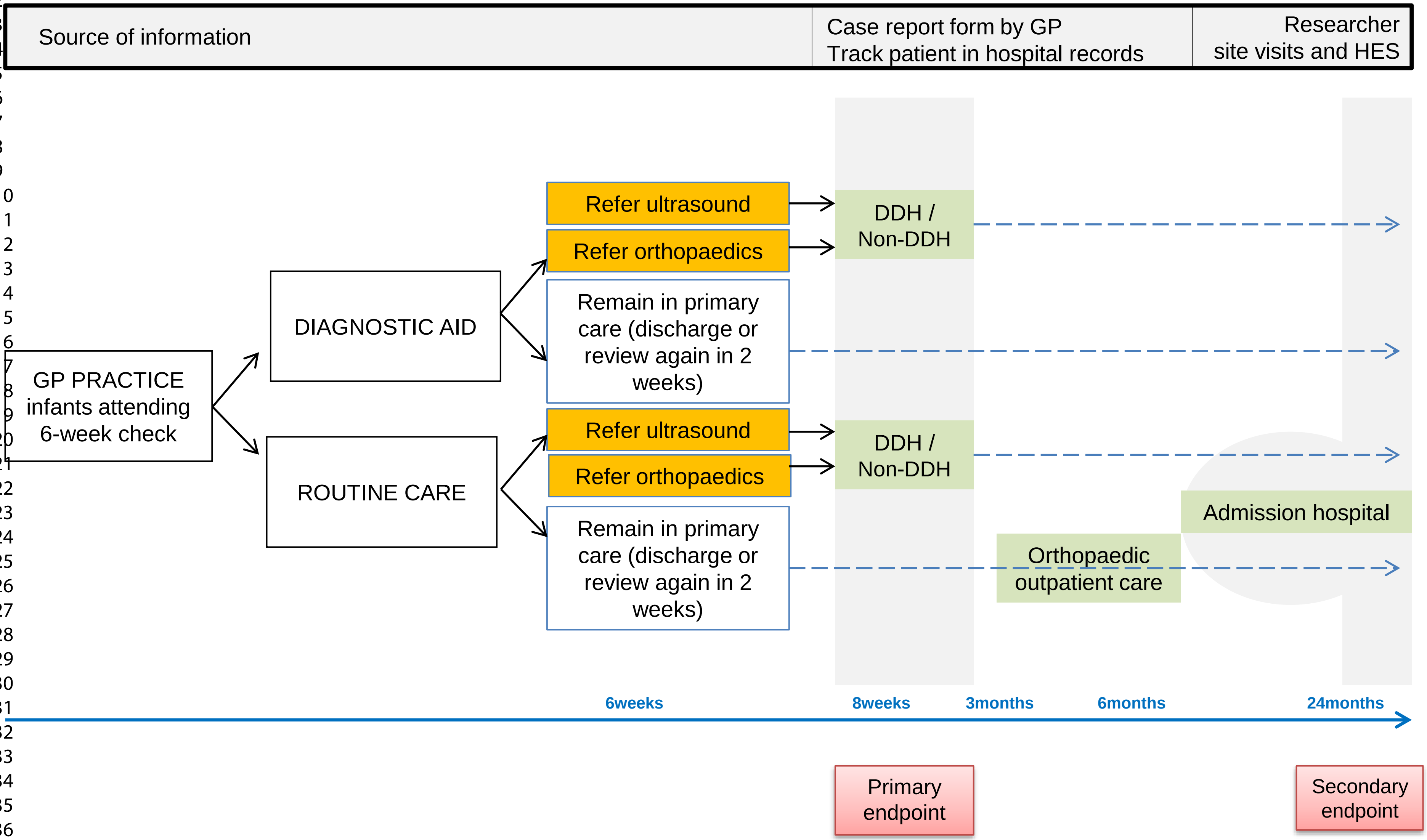
Source of information
Case report form by GP
Track patient in hospital records
Researcher
site visits and HES
Refer ultrasound
Refer orthopaedics
DDH / Non-DDH
DIAGNOSTIC AID
Remain in primary care (discharge or review again in 2 weeks)
GP PRACTICE infants attending 6-week check
Refer ultrasound
Refer orthopaedics
DDH / Non-DDH
ROUTINE CARE
Admission hospital
Remain in primary care (discharge or review again in 2 weeks)
Orthopaedic outpatient care
6weeks
8weeks
3months
6months
24months
Primary endpoint
Secondary endpoint

Appendix 1. ROLES AND RESPONSIBILITIES

Funder

The funding body had or has no involvement in study design; collection, management, analysis and interpretation of data; or the decision to submit for publication. The funding body will be informed of any planned publications, and documentation provided.

Sponsor

The sponsor for this trial is Great Ormond Street Hospital for Children. The sponsor is responsible for providing the investigator with the necessary information to conduct the clinical trial, to ensure proper monitoring of the trial and ensuring compliance to ethical bodies and legislation. The sponsor works to the UK Policy Framework for Health and Social Care Research. The sponsor is not involved in aspects of study design, report writing or data analysis. They are the data controller and all data shall return to the sponsor at the end of the trial. A collaboration agreement is in place with all organisations of the co-investigators. Data processing agreements are in place for situations where data will be collected and processed outside of Great Ormond Street Hospital. The sponsor can be contacted by email (research.governance@gosh.nhs.uk) or telephone 0207 905 2249.

Coordinating centre

PRIMENT Clinical Trials Unit is coordinating this trial. A trial management group has been set up within PRIMENT for the monthly monitoring of the trial conduct. It includes the chief investigator, director of the trials unit, programme manager and trial manager. PRIMENT are responsible for overseeing the conduct and progress of the trial.

Steering committee

The steering committee includes an independent chair, two further independent members (one is a biostatistician), sponsor representative, funder representative, chief investigator and two further members of the research team including. The committee will provide overall

For peer review only

supervision of the trial, review analysis plan, recruitment figures and outcome data, and provide recommendations for any appropriate actions for the trial as necessary.

Data monitoring committee

The steering committee will take on the role of the data monitoring committee.

Data Access

All identifiable data will be stored on encrypted servers, UCL Data Safe Haven. Only restricted members of the research team will be able to access this data. De-identified data will be shared with the wider members of the research team.

For peer review only

Reporting checklist for protocol of a clinical trial.

Based on the SPIRIT guidelines.

Instructions to authors

Complete this checklist by entering the page numbers from your manuscript where readers will find each of the items listed below.

Your article may not currently address all the items on the checklist. Please modify your text to include the missing information. If you are certain that an item does not apply, please write "n/a" and provide a short explanation.

Upload your completed checklist as an extra file when you submit to a journal.

In your methods section, say that you used the SPIRITreporting guidelines, and cite them as:

Chan A-W, Tetzlaff JM, Altman DG, Laupacis A, Gøtzsche PC, Krleža-Jerić K, Hróbjartsson A, Mann H, Dickersin K, Berlin J, Doré C, Parulekar W, Summerskill W, Groves T, Schulz K, Sox H, Rockhold FW, Rennie D, Moher D. SPIRIT 2013 Statement: Defining standard protocol items for clinical trials. Ann Intern Med. 2013;158(3):200-207

		Reporting Item	Page Number
**Administrative information**			
Title	#1	Descriptive title identifying the study design, population, interventions, and, if applicable, trial acronym	1
Trial registration	#2a	Trial identifier and registry name. If not yet registered, name of intended registry	3
Trial registration: data set	#2b	All items from the World Health Organization Trial Registration Data Set	3
Protocol version	#3	Date and version identifier	15
Funding	#4	Sources and types of financial, material, and other support	16
Roles and responsibilities: contributorship	#5a	Names, affiliations, and roles of protocol contributors	1

For peer review only

Roles and responsibilities: sponsor contact information	#5b	Name and contact information for the trial sponsor	Appendix 1
Roles and responsibilities: sponsor and funder	#5c	Role of study sponsor and funders, if any, in study design; collection, management, analysis, and interpretation of data; writing of the report; and the decision to submit the report for publication, including whether they will have ultimate authority over any of these activities	Appendix 1
Roles and responsibilities: committees	#5d	Composition, roles, and responsibilities of the coordinating centre, steering committee, endpoint adjudication committee, data management team, and other individuals or groups overseeing the trial, if applicable (see Item 21a for data monitoring committee)	Appendix 1
**Introduction**			
Background and rationale	#6a	Description of research question and justification for undertaking the trial, including summary of relevant studies (published and unpublished) examining benefits and harms for each intervention	4
Background and rationale: choice of comparators	#6b	Explanation for choice of comparators	5
Objectives	#7	Specific objectives or hypotheses	6
Trial design	#8	Description of trial design including type of trial (eg, parallel group, crossover, factorial, single group), allocation ratio, and framework (eg,	6

For peer review only

		superiority, equivalence, non-inferiority, exploratory)	
**Methods: Participants, interventions, and outcomes**			
Study setting	#9	Description of study settings (eg, community clinic, academic hospital) and list of countries where data will be collected. Reference to where list of study sites can be obtained	7
Eligibility criteria	#10	Inclusion and exclusion criteria for participants. If applicable, eligibility criteria for study centres and individuals who will perform the interventions (eg, surgeons, psychotherapists)	7
Interventions: description	#11a	Interventions for each group with sufficient detail to allow replication, including how and when they will be administered	7
Interventions: modifications	#11b	Criteria for discontinuing or modifying allocated interventions for a given trial participant (eg, drug dose change in response to harms, participant request, or improving / worsening disease)	N/A. The experimental intervention (diagnostic aid) is an electronic tool that cannot be modified. If a practice wishes to withdraw from its use, it can do at any point of the trial. Parent/carers can opt out of its use and any data collection at the 6 week check.
Interventions: adherance	#11c	Strategies to improve adherence to intervention protocols, and any procedures for monitoring adherence (eg, drug tablet return; laboratory tests)	7

For peer review only

Interventions: concomitant care	#11d	Relevant concomitant care and interventions that are permitted or prohibited during the trial	N/A. The experimental intervention (diagnostic aid) is a low risk electronic checklist and does not affect any concomitant care
Outcomes	#12	Primary, secondary, and other outcomes, including the specific measurement variable (eg, systolic blood pressure), analysis metric (eg, change from baseline, final value, time to event), method of aggregation (eg, median, proportion), and time point for each outcome. Explanation of the clinical relevance of chosen efficacy and harm outcomes is strongly recommended	9
Participant timeline	#13	Time schedule of enrolment, interventions (including any run-ins and washouts), assessments, and visits for participants. A schematic diagram is highly recommended (see Figure)	Table 1
Sample size	#14	Estimated number of participants needed to achieve study objectives and how it was determined, including clinical and statistical assumptions supporting any sample size calculations	8
Recruitment	#15	Strategies for achieving adequate participant enrolment to reach target sample size	7
**Methods: Assignment of interventions (for controlled trials)**			

For peer review only

Allocation: sequence generation	#16a	Method of generating the allocation sequence (eg, computer-generated random numbers), and list of any factors for stratification. To reduce predictability of a random sequence, details of any planned restriction (eg, blocking) should be provided in a separate document that is unavailable to those who enrol participants or assign interventions	7
Allocation concealment mechanism	#16b	Mechanism of implementing the allocation sequence (eg, central telephone; sequentially numbered, opaque, sealed envelopes), describing any steps to conceal the sequence until interventions are assigned	7
Allocation: implementation	#16c	Who will generate the allocation sequence, who will enrol participants, and who will assign participants to interventions	7
Blinding (masking)	#17a	Who will be blinded after assignment to interventions (eg, trial participants, care providers, outcome assessors, data analysts), and how	15
Blinding (masking): emergency unblinding	#17b	If blinded, circumstances under which unblinding is permissible, and procedure for revealing a participant's allocated intervention during the trial	N/A. Trial participants will not be blinded.
**Methods: Data collection, management, and analysis**			
Data collection plan	#18a	Plans for assessment and collection of outcome, baseline, and other trial	9

For peer review only

		data, including any related processes to promote data quality (eg, duplicate measurements, training of assessors) and a description of study instruments (eg, questionnaires, laboratory tests) along with their reliability and validity, if known. Reference to where data collection forms can be found, if not in the protocol	
Data collection plan: retention	#18b	Plans to promote participant retention and complete follow-up, including list of any outcome data to be collected for participants who discontinue or deviate from intervention protocols	7
Data management	#19	Plans for data entry, coding, security, and storage, including any related processes to promote data quality (eg, double data entry; range checks for data values). Reference to where details of data management procedures can be found, if not in the protocol	14
Statistics: outcomes	#20a	Statistical methods for analysing primary and secondary outcomes. Reference to where other details of the statistical analysis plan can be found, if not in the protocol	11
Statistics: additional analyses	#20b	Methods for any additional analyses (eg, subgroup and adjusted analyses)	11
Statistics: analysis population and missing data	#20c	Definition of analysis population relating to protocol non-adherence (eg, as randomised analysis), and any statistical methods to handle missing data (eg, multiple imputation)	12

For peer review only

**Methods: Monitoring**			
Data monitoring: formal committee	#21a	Composition of data monitoring committee (DMC); summary of its role and reporting structure; statement of whether it is independent from the sponsor and competing interests; and reference to where further details about its charter can be found, if not in the protocol. Alternatively, an explanation of why a DMC is not needed	Appendix 1
Data monitoring: interim analysis	#21b	Description of any interim analyses and stopping guidelines, including who will have access to these interim results and make the final decision to terminate the trial	N/A. No interim analysis has been planned for this trial.
Harms	#22	Plans for collecting, assessing, reporting, and managing solicited and spontaneously reported adverse events and other unintended effects of trial interventions or trial conduct	16
Auditing	#23	Frequency and procedures for auditing trial conduct, if any, and whether the process will be independent from investigators and the sponsor	N/A. We have no plans for auditing this trial at the moment. the sponsor may conduct an audit at a random time. The clinical trial unit will conduct monthly central monitoring visits.
**Ethics and dissemination**			
Research ethics approval	#24	Plans for seeking research ethics committee / institutional review board (REC / IRB) approval	15

For peer review only

Protocol amendments	#25	Plans for communicating important protocol modifications (eg, changes to eligibility criteria, outcomes, analyses) to relevant parties (eg, investigators, REC / IRBs, trial participants, trial registries, journals, regulators)	16 and Appendix 1
Consent or assent	#26a	Who will obtain informed consent or assent from potential trial participants or authorised surrogates, and how (see Item 32)	7
Consent or assent: ancillary studies	#26b	Additional consent provisions for collection and use of participant data and biological specimens in ancillary studies, if applicable	N/A. No collection of samples in this trial.
Confidentiality	#27	How personal information about potential and enrolled participants will be collected, shared, and maintained in order to protect confidentiality before, during, and after the trial	9
Declaration of interests	#28	Financial and other competing interests for principal investigators for the overall trial and each study site	16
Data access	#29	Statement of who will have access to the final trial dataset, and disclosure of contractual agreements that limit such access for investigators	Appendix 1
Ancillary and post trial care	#30	Provisions, if any, for ancillary and post-trial care, and for compensation to those who suffer harm from trial participation	N/A. No post trial care is expected. The respective information sheets contain details of Patient Advice Liasion (PALs) services that participants can contact for complaints and compensation.

For peer review only

Dissemination policy: trial results	#31a	Plans for investigators and sponsor to communicate trial results to participants, healthcare professionals, the public, and other relevant groups (eg, via publication, reporting in results databases, or other data sharing arrangements), including any publication restrictions	15
Dissemination policy: authorship	#31b	Authorship eligibility guidelines and any intended use of professional writers	N/A. These have not yet been drawn up.
Dissemination policy: reproducible research	#31c	Plans, if any, for granting public access to the full protocol, participant-level dataset, and statistical code	15
**Appendices**			
Informed consent materials	#32	Model consent form and other related documentation given to participants and authorised surrogates	N/A. This trial is multifaceted and thus has many information sheets and consent forms for different aspects of the trial.
Biological specimens	#33	Plans for collection, laboratory evaluation, and storage of biological specimens for genetic or molecular analysis in the current trial and for future use in ancillary studies, if applicable	N/A. No collection of samples required for this trial.

None The SPIRIT checklist is distributed under the terms of the Creative Commons Attribution License CC-BY-ND 3.0. This checklist can be completed online using https://www.goodreports.org/, a tool made by the EQUATOR Network in collaboration with Penelope.ai

For peer review only
